# Interaction between Copper Oxide Nanoparticles and Amino Acids: Influence on the Antibacterial Activity

**DOI:** 10.3390/nano9050792

**Published:** 2019-05-23

**Authors:** Elena Badetti, Loris Calgaro, Laura Falchi, Alessandro Bonetto, Cinzia Bettiol, Benedetta Leonetti, Emmanuele Ambrosi, Elisabetta Zendri, Antonio Marcomini

**Affiliations:** 1 DAIS—Department of Environmental Sciences, Informatics and Statistics, University Ca’ Foscari of Venice, Via Torino 155, 30172 Venice Mestre, Italy; loris.calgaro@unive.it (L.C.); laura.falchi@unive.it (L.F.); alessandro.bonetto@unive.it (A.B.); bettiol@unive.it (C.B.); elizen@unive.it (E.Z.); 2 DMSN—Department of Molecular Sciences and Nanosystems, University Ca’ Foscari of Venice, Via Torino 155/b, 30172 Venice Mestre, Italy; leonetti393@gmail.com (B.L.); emmanuele.kizito@gmail.com (E.A.); 3 ECLT Lab—European Centre for Living Technology, University Ca’ Foscari of Venice, Via Torino 155/b, 30172 Venice Mestre, Italy

**Keywords:** copper oxide nanoparticles, amino acids, antimicrobial activity

## Abstract

The increasing concern about antibiotic-resistance has led to the search for alternative antimicrobial agents. In this effort, different metal oxide nanomaterials are currently under investigation, in order to assess their effectiveness, safety and mode of action. This study focused on CuO nanoparticles (CuO NPs) and was aimed at evaluating how the properties and the antimicrobial activity of these nanomaterials may be affected by the interaction with ligands present in biological and environmental media. Ligands can attach to the surface of particles and/or contribute to their dissolution through ligand-assisted ion release and the formation of complexes with copper ions. Eight natural amino acids (L-Arg, L-Asp, L-Glu, L-Cys, L-Val, L-Leu, L-Phe, L-Tyr) were chosen as model molecules to investigate these interactions and the toxicity of the obtained materials against the Gram-positive bacterium *Staphylococcus epidermidis* ATCC 35984. A different behavior from pristine CuO NPs was observed, depending on the aminoacidic side chain. These results were supported by physico-chemical and colloidal characterization carried out by means of Fourier-Transform Infrared spectroscopy (FTIR), Differential Scanning Calorimetry (DSC) and Thermo-Gravimetric Analysis (TGA), Inductively Coupled Plasma Mass Spectrometry (ICP-MS) and light scattering techniques (Dynamic Light Scattering (DLS), Electrophoretic Light Scattering (ELS) and Centrifugal Separation Analysis (CSA).

## 1. Introduction

In the last decades, nanotechnology has drawn increasing attention in various fields of research, such as environmental sciences, pharmacology, medicine and microbiology. Among the wide variety of nanomaterials available, metal and metal oxide nanoparticles are nowadays used in a number of industrial and consumer applications, and some of them are attracting great interest specifically owing to their bactericidal properties [1,2,3,4]. The well-known problem of resistance of pathogenic bacteria to conventional antibiotics and the emergence of multidrug-resistant strains has prompted the search for novel antimicrobial agents. In this perspective, nano Ag has been deeply studied, and is currently employed in different consumer and medical products [5,6,7,8]. However, more recent research has focused on copper nanomaterials, based on the long-known antifungal and antibacterial properties of Cu compounds, which are widely used e.g., in agriculture and in antifouling paints [9,10,11]. This, together with the lower cost of copper compared to silver [7], makes Cu-based nanoparticles promising candidates for the development of new antimicrobial agents to control resistant bacterial pathogens.

Though in the last few years the study of these nanomaterials for use in this application is rapidly increasing, they are still poorly understood in terms of behavior, fate and toxic action in complex media, which can ultimately influence their effectiveness and safety [12].

The antibacterial activity of CuO nanoparticles (CuO NPs) is generally attributed to both the released Cu(II) ions and the particles themselves, but uncertainties remain about their mechanism of toxic action, which is supposed to be exerted mainly through oxidative stress induced by reactive oxygen species (ROS) generation, disruption of cell wall and membrane, enzymatic inhibition and DNA damage [1,3,7].

Compared to the bulk counterpart, Cu-based nanoparticles may undergo profound transformations, under different biological and environmental conditions, which can induce significant changes in their structural and physico-chemical properties and thus, in turn, affect their toxicity [13,14]. Specifically, the interaction with other molecules present in biological and environmental media may greatly influence particle solubility, aggregation status and surface properties [15].

This study aims at evaluating how the structural and physico-chemical properties and the antimicrobial activity of CuO NPs may be affected by the interactions between these nanomaterials and a series of natural amino acids. The latter were chosen as model molecules because of their importance in biological systems. These ligands, which can be present in biological and environmental media, can attach to the surface of CuO nanoparticles and/or contribute to their dissolution through ligand-assisted ion release, thus inducing changes both in the type and extent of toxic effects. The role played by different aminoacidic side chains was evaluated by means of Fourier-Transform Infrared spectroscopy (FTIR), Differential Scanning Calorimetry (DSC) and Thermo-Gravimetric Analysis (TGA), Inductively Coupled Plasma Mass Spectrometry (ICP-MS) and light scattering techniques, applied to both pristine and amino acid-treated CuO NPs. To further investigate these interactions, the corresponding Cu-amino acid complexes were also synthetized and characterized by FTIR and ICP-MS. The information gathered from these analyses was used to interpret the results of a toxicity test performed against the Gram-positive bacterium *Staphylococcus epidermidis* ATCC 35984.

## 2. Materials and Methods

### 2.1. Materials

Commercial CuO nanopowder (average particle diameter: 12 ± 4 nm) was provided by PlasmaChem GmbH (Berlin, Germany). Ultrapure H_2_O_2_ and HNO_3_ for trace metal analysis were purchased from AppliChem GmbH (Darmstadt, Germany). Amino acids, CuCl_2_, CuSO_4_, NaOH, EtOH, Nutrient Broth (NB) and Nutrient Agar (NA) were purchased from Merck KGaA (Darmstadt, Germany). The used bacterial strain was *Staphylococcus epidermidis* ATCC 35984. Ultrapure water with a resistivity of 18.2 MΩcm was obtained with MilliQ system from Merck KGaA (Darmstadt, Germany).

### 2.2. Treatment of CuO NPs with Amino Acids

**Figure 1:**
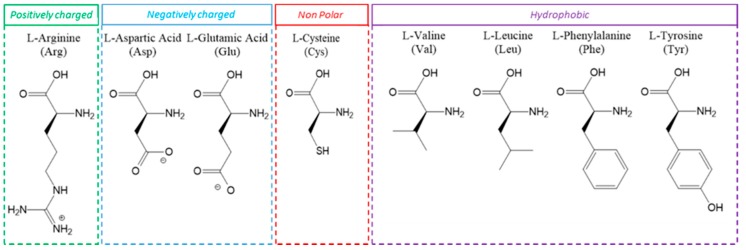
Chemical structure of the selected amino acids grouped according to the chemical characteristics of the side chain.

About 500 mg (6.3 mmol) of CuO NPs in 100 mL of ultrapure water were sonicated with an ultrasonic probe UP-200S by Hielscher Ultrasonics GmbH (Teltow, Germany) for 15 min in an ice bath (200 W, pulsed 80% mode). The amino acids reported in Figure 1, L-Arg, L-Asp, L-Glu, L-Cys, L-Val, L-Leu, L-Phe, L-Tyr (6.3 mmol in 400 mL of ultrapure water), were added to the dispersed NPs and the resulting suspensions (pH of around 6) were probe sonicated for 1 h and then magnetically stirred overnight at room temperature. Afterwards, each suspension was centrifuged for 15 min at 8000 rpm (8014 RCF). The supernatant was removed, and the pellets were washed by adding 150 mL of ultrapure water, followed by ultra-sonication and centrifugation. This washing procedure was repeated twice, once with water and once with EtOH, to remove the excess of unreacted amino acid. After the last washing step, the pellets were dried, leading to powders that were physico-chemically characterized as described below and used to perform tests on bacteria. Powders yield were calculated as *w/w* and resulted in ~30% for CuO NPs treated with Arg and Val, 96% for CuO NPs treated with Cys and ~98% for CuO NPs treated with Asp, Glu, Leu, Phe and Tyr.

### 2.3. Synthesis of Cu-Amino Acid Complexes

Copper(II) complexes with Asp, Glu, Leu, Phe and Tyr were prepared adapting the experimental procedure reported by Stanila et al. [16] for Cu-(Phenilalanine)_2_ 2H_2_O complex. In detail, about 3 mmol of each amino acid (0.399 g of Asp, 0.441 g of Glu, 0.393 g of Leu, 0.495 g of Phe and 0.543 g of Tyr) were dissolved in ultrapure water (275 mL for Asp, 90 mL for Glu, 20 mL for Val, 0.393 mL for Leu, 160 mL for Phe and 200 mL for Tyr), and 0.33 mL of a 30% NaOH solution were added. Then, 1.5 mmol of CuCl_2_ were dissolved in 4 mL of ultrapure water and added to each amino acid solution under stirring. The precipitation was instantaneous for all amino acids, except for Glu and Tyr, which required to be concentrated up to 20 mL under vacuum. Each precipitate was centrifuged for 15 min at 8000 rpm (8014 RCF). The supernatant was removed, and the complexes were washed twice with 40 mL of ultrapure water. After washing, each compound was vacuum dried and characterized by means of FTIR and ICP-MS, as described below. The final yield was about 79% for Asp, 56% for Glu, 51% for Leu, 57% for Phe and 44% for Tyr.

The Cu-Arg complex was prepared by dissolving 2 mmol of Arg (0.352 g) in 10 mL of ultrapure water and adding to this solution 1 mmol of CuCl_2_ (0.134 g) dissolved in 10 mL of ultrapure water [17]. The reaction mixture was heated overnight at 70 °C and then cooled at room temperature. The final volume of water was reduced to favor the precipitation of the desired complex. The solid obtained by centrifugation was washed, dried (yield 65%) and characterized.

The Cu-Cys complex was prepared by dissolving 2 mmol of Cys (0.242 g) and 1 mmol of CuCl_2_ (0.134 g) in 50 mL of EtOH [18]. The reaction was left stirring at room temperature for 30 min. A brown solid precipitate was formed and recovered by centrifugation. The solid obtained (yield 78%) was dried and characterized.

The Cu-Val complex was not synthetized since it could be recovered from the supernatant obtained from the treatment of CuO-NPs with Val described above, after centrifugation. Blue crystals of the complex precipitated from the supernatant after 2 weeks (yield 68%).

### 2.4. FTIR and TGA-DSC

The physico-chemical characterization on the dry powders was performed by means of Fourier-Transform Infrared spectroscopy (FTIR), Thermo-Gravimetric Analysis (TGA) and Differential Scanning Calorimetry (DSC). This kind of analysis has already been successfully used in a previous study with TiO_2_ NPs functionalized with catechol derivatives, to estimate the NPs surface coverage rate for each ligand [19].

In detail, Fourier Transform Infrared spectroscopy (FTIR) analysis was performed with a Thermo Nicolet Nexus 670 FTIR spectrophotometer (Thermo Fisher Scientific, Waltham, MA, USA) equipped with a Smart Orbit Single Reflection Diamond ATR (Attenuated Total Reflection) accessory, from 4000 to 400 cm^−1^ for 64 scans with 4 cm^−1^ resolution. FTIR data were elaborated with Omnic (version 8.0, Thermo Fisher Scientific, Waltham, MA, USA) and Origin software (version 8.5, OriginLab, Northampton, MA, USA).

Thermo-Gravimetric Analysis (TGA) and Differential Scanning Calorimetry (DSC) were performed simultaneously using a Netzsch 409/C apparatus (Netzsch-Gruppe, Selb, Germany). The temperature program used was set up experimentally from 30 to 600 °C, with a heating rate of 10 °C min^−1^. Samples (around 15 mg) were placed in a platinum/rhodium crucible and alumina was used for the internal calibration. Measurements were performed in air/N_2_ (40/80 mL/min) mixture. Data were collected with STA Netzsch software (Netzsch-Gruppe, Selb, Germany) and then elaborated with Origin 8.5 software.

### 2.5. ICP-MS

The total content of copper in the dry powders was determined by Inductively Coupled Plasma Mass Spectrometry (ICP-MS NexION 350D, Perkin Elmer, Waltham, MA, USA). Before ICP-MS analysis, samples were microwave digested by using a Discover SP-D oven (CEM Corporation, Matthews, NC, USA). In 35 mL glass vessels, dry samples (about 20 mg) were treated with 2 mL ultrapure H_2_O_2_ and 4 mL ultrapure HNO_3_. The heating program used for the acid digestion was: T_MAX_ = 170 °C, Ramp Time = 5 min, Hold Time = 2 min, Power = 300 W. Afterwards, the samples were cooled down for 30 min at room temperature and made up to volume with ultrapure water. After appropriate dilutions, samples were analyzed by ICP-MS, by using the parameters reported in Table 1. Copper content in samples was quantified by external calibration method using a multi-point curve (five points over the concentration range 0.5 to 10 mg/L of copper). Yttrium at 5 µg/L was used as internal standard. Reagent blanks were included in the analysis. Measurements were performed in triplicate.

### 2.6. SEM

The surface topography of CuO nanoparticles was investigated by Scanning Electron Microscopy (SEM). The dry samples were suspended in EtOH (0.1–0.5 mg/mL final concentration) and briefly ultrasonicated. About 3 μL of the suspension were deposited on a silicon wafer substrate and dried at 60 °C for 18–24 h. Images were collected in high vacuum with a Zeiss Sigma VP Field Emission SEM (Carl Zeiss, Oberkochen, Germany), using an in-lens detector at 5.0 keV beam energy.

### 2.7. DLS, ELS and CSA

The colloidal characterization of the dispersions was assessed by Dynamic Light Scattering (DLS) and Electrophoretic Light Scattering (ELS) by means of a multi-angle Nicomp ZLS Z3000 (Particle Sizing System, Port Richey, FL, USA). The dried powders were re-dispersed in ultrapure water (50 mg/L) by probe sonication in an ice bath at 200 W for 5 min (in pulsed 80% mode). All measurements were taken after a pre-equilibration of ca. 10 min.

In detail, the hydrodynamic diameter was measured with an optical fiber set at 90° scattering angle (W = 25 mW and λ = 639 nm) over at least 6 min at room temperature. Surface charge of the electric double layer of each sample was determined by applying a 5 V electric field to obtain zeta-potential (ζ-pot) values. Sedimentation velocity was determined through Centrifugal Separation Analysis (CSA), by using a Multiwavelength Dispersion Analyzer LUMiSizer^®^ 651 (Lum GmbH, Berlin, Germany). The transmission profiles obtained by CSA (transmittance values over the length of the cuvette filled with the sample) are due to the particles’ migration, caused by the centrifugal force [20]. Analyses were performed at 4000 rotations per minute (rpm), which corresponds to a relative centrifugal force (rcf) of 2146 at 120 mm from the rotor of the centrifuge. Sedimentation velocity data were calculated from the transmittance values obtained setting the wavelength of the transmitted light at 470 nm and collecting the transmittance (%) over time at three different positions (115, 120 and 125 mm from the rotor) over the length of the cuvette. The runtime of each analysis (25 min) was selected to reach the complete sedimentation of NPs. Sedimentation velocity data at gravity were calculated by dividing the obtained values for the applied rcf. Hydrodynamic diameter, surface charge and sedimentation velocity were measured in duplicate and the results were reported as average for DLS/ELS and as median for CSA.

### 2.8. Antibacterial Test

Antibacterial tests were conducted by plating *S. epidermidis* on Nutrient Agar (NA) containing a range of concentrations of the different materials to be tested (pristine and treated CuO NPs, Cu-amino acid complexes, amino acids and Cu salts). For each material, agar plates were prepared by adding appropriate amounts of a stock suspension or solution to nutrient agar (allowed to cool to 70 °C), to reach final concentrations of 31.3, 62.5, 125, 250 and 500 μg/mL. Plates for negative controls were prepared without any addition of antibacterial agents. *S. epidermidis* ATCC 35984 was inoculated into Nutrient Broth (NB) and grown overnight at 37 °C. After OD_600_ measurement and appropriate dilution, about 400 Colony Forming Units (CFUs) were plated and grown overnight at 37 °C. Pictures of plates were acquired with Geliance 600 Images System and colonies were counted using ImageJ software (National Institute of Health, Bethesda, MD, USA). The Minimal Inhibitory Concentration (MIC) is defined as the lowest concentration of material at which no visible colonies of microorganism are observed. All experiments were conducted in both biological and technical triplicate.

## 3. Results and Discussion

The dynamic nature of CuO NPs and consequently the profound transformations to which they can be subjected when dispersed in media containing other chemicals are well known. In this specific case, amino acids are known to form high-affinity complexes with copper, with generally very high stability constants [21]. This means that, in the presence of CuO NPs, amino acids can: (i) get attached on the NP surface; (ii) remove ions from the surface after complex formation; (iii) form complexes by removing the free ions from the ion-particle equilibrium. These last two mechanisms favor the dissolution of nanoparticles to different degrees, depending on the nature of the amino acid. In general, these kinetic differences are associated with ligand-specific surface binding ability [22]. As reported in the literature, the amino and carboxylate groups are mainly involved in the complexes’ formation [23], with some exceptions, in which also the side chain is involved. The nature of the aminoacidic chain is also responsible for the formation of soluble or insoluble complexes, which can also influence the nanoparticles/complex equilibrium.

Based on these evidences, the data obtained from the application of the different analytical techniques to the materials derived from the treatment of CuO NPs with amino acids, as well as to the corresponding Cu-amino acid complexes, will be discussed below.

### 3.1. ATR-FTIR

**Figure 2:**
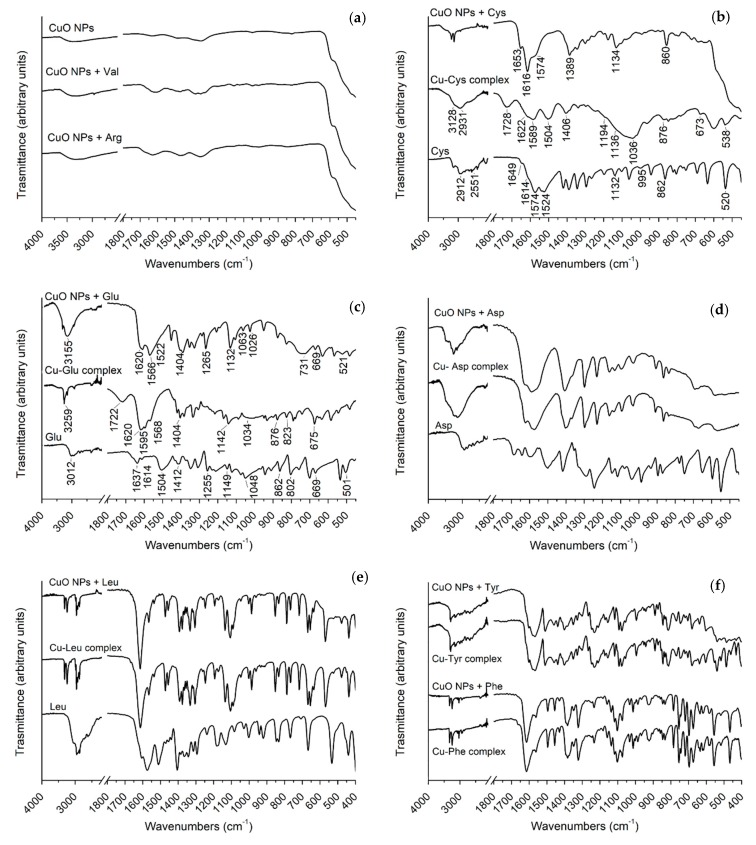
Fourier Transform Infrared spectroscopy (FTIR) spectra of (**a**) CuO nanoparticles (CuO NPs) pristine and treated with Arg and Val; (**b**) free Cys, Cu-Cys complex and CuO NPs treated with Cys; (**c**) free Glu, Cu-Glu complex and CuO NPs treated with Glu (**d**) free Asp, Cu-Asp complex and CuO NPs treated with Asp; (**e**) free Leu, Cu-Leu complex and CuO NPs treated with Leu; (**f**) Cu-Phe complex, CuO NPs treated with Phe, Cu-Tyr complex and CuO NPs treated with Tyr.

The behavior of the different amino acids with respect to CuO NPs was firstly evaluated by FTIR (Figure 2). The spectrum of pristine CuO NPs showed a main band in the region around 500 cm^−1^, related to the stretching Cu–O–Cu, while the broad bands observed in the region 1200–1600 cm^−1^ and at 3000 cm^−1^ can be attributed to –OH groups and to residual organic components (i.e., acetates) used in their synthesis [24].

CuO NPs treated with Val and Arg presented the same FTIR spectra as pristine NPs (Figure 2a, Figures S1 and S2). Considering the low amount of solid recovered after the washing steps (~30%) and the blue color of the supernatant, it can be reasonably assumed that water-soluble copper(II)-amino acid complexes were formed and removed, leaving in the pellets only non-solubilized CuO NPs. In fact, crystals of Cu(II)-valine complex were isolated from the supernatant after 2 weeks, and the corresponding FTIR spectrum is reported in Figure S1. This FTIR spectrum, as well as that of Cu-Arg complex (synthetized as described in the experimental section), are in accordance to those reported in the literature [17,25].

This suggests that both Val and Arg, with a hydrophobic and a positive side chain respectively, do not remain covalently attached on the CuO NPs surface but form soluble Cu(II) complexes by removing copper from CuO NPs. This process favors the dissolution of CuO NPs, which occurs following the two dissolution mechanisms proposed above. The molar ratio between NPs and amino acids, the reaction time and the sonication power are the parameters that mainly control the amount of complexes formed and consequently the degree of CuO NPs’ dissolution.

For the other amino acids, the materials obtained always showed spectra with stretching and bending bands that differ from those of both pristine NPs and the free amino acids (Figure 2, and Figures S3–S9). The possible bonding on NPs’ surface or the formation of Cu(II) complexes, insoluble in both water and EtOH, were investigated by comparing the spectra of the final nanomaterials with those of the corresponding Cu(II)-amino acid complexes.

In detail, with respect to the free amino acid, a loss of the IR hyperfine structure for both CuO NPs treated with Cys and the Cu-Cys complex was observed (Figure 2b and Figure S3). The comparison among the three spectra highlighted the following major differences: (i) in the 3500–2000 cm^−1^ range, where free Cys showed a complex pattern (N–H stretching extended by combination of overtones bands superimposed on aliphatic C–H stretch), the Cu-Cys complex showed a less refined structure, while CuO NPs treated with Cys presented only few well-defined peaks with low intensity. Moreover, the strong vibration of S–H observed for the free amino acid (at 2551 cm^−1^) totally disappeared in both the treated CuO NPs and the complex, probably due to Cu-S bonding; (ii) in the 1800–1450 cm^−1^ range, the signals of treated CuO NPs (i.e., the stretching of the carboxylate group and bending of amine) were shifted towards higher wavenumbers (by about 50–100 cm^−1^) with respect to free Cys, with changes in the relative intensities. In this region, differently from CuO NPs treated with Cys, the complex showed the carbonyl stretching at 1728 cm^−1^; (iii) below 1200 cm^−1^, the –SH bending signal of free Cys (at 995 cm^−1^) disappeared for both treated CuO NPs and the complex. The COO^−^ bending of free Cys (at 520 cm^−1^) and of Cu-Cys complex (at 538 cm^−1^) was instead covered for the treated CuO NPs by the stretching signals of Cu–O–Cu. The bands assigned to –NH_3_^+^ and C–N (at 1132 and 1063 cm^−1^ respectively), which were well defined in the free Cys, were merged in CuO NPs treated with Cys, while they appeared as a broad signal in the case of the Cu-Cys complex. Finally, the signal corresponding to –COO^−^ bending for Cys (at around 860 cm^−1^) was in the same position for treated CuO NPs, but it was shifted to 876 cm^−1^ and much less intense for the corresponding complex.

These findings suggest that in the Cu-Cys complex, according to the literature [18], Cys is probably coordinated to the Cu(II) only by the thiol group. On the other side, in CuO NPs treated with Cys, not only the –SH but also the –NH_2_ and COOH groups are likely to be involved in the interaction between the nanoparticles and Cys. Therefore, it is reasonable to assume that Cys is bonded to the surface of CuO NPs, confirming what was already reported by Peng et al. for similar materials [26].

Similar to Cys, CuO NPs treated with Glu (negative side chain) showed a different FTIR spectrum from both Cu-Glu complex and free Glu (Figure 2c). As described in the literature for the Cu-Glu complex [27], Glu is most likely coordinated to Cu(II) by the α-COO^−^ and -NH_2_ groups, as can be argued from the shift of the signals of Glu at around 860 and 1048 cm^−1^. Moreover, the presence of a signal at 1722 cm^−1^, corresponding to C=O stretching, indicated the protonation of the side chain carboxylate group. On the other side, in the spectrum of CuO NPs treated with Glu, the absence of this carbonyl stretching in the 1700–1790 cm^−1^ region suggests that the γ-carboxylate group is involved in the interaction between the amino acid and copper, together with α-COO^−^ and NH_2_ groups. As in the case of Cys, we can thus infer that also Glu is bonded to the CuO NPs surface; in addition, both amino acids are bonded to NPs in a different way with respect to the corresponding complexes in terms of stoichiometry (Cu:amino acid) and functional groups involved in the coordination with copper.

Surprisingly, CuO NPs treated with Asp, also bearing a negative side chain and with a structure quite similar to Glu, showed a very similar FTIR spectrum than the corresponding Cu-Asp complex (Figure 2d and Figure S5). This probably indicates the presence of the complex (insoluble in both water and EtOH) in the material obtained from the treatment of CuO NPs with Asp.

In the same way, the FTIR spectra of CuO NPs treated with amino acids with a hydrophobic side chain, i.e., Leu, Phe and Tyr (Figure 2e–f and Figures S6–S8), showed an absorption pattern analogous to those of the corresponding Cu-amino acid complexes, which also correspond to those reported in the literature [28].

In addition, the stretching band of Cu–O–Cu around 500 cm^−1^ was observed in the FTIR spectra of the CuO NPs treated with Leu and Tyr, while in the materials containing Asp and Phe this band was not clearly distinguishable (probably convoluted with signals ascribable to Cu-complexes).

The overall results suggest that only Cys and Glu were bonded on CuO NPs surface, while the other amino acids, independently from the nature of the side chain, favored CuO NPs dissolution through ligand-assisted ion release, forming Cu(II)-amino acid complexes. In detail, while Val and Arg formed Cu(II) complexes that were soluble in water, Asp, Leu, Phe and Tyr seemed to form insoluble complexes which were not removed from the final material in the washing steps with water and EtOH (Figure 2e–f and Figures S5–S8), leading to physical mixtures of CuO NPs and Cu-amino acid complexes.

To confirm that Cys and Glu are attached on the CuO NPs surface, two physical mixtures were prepared by mixing in a mortar pristine CuO NPs with Cu-Cys and Cu-Glu complexes (ratio of 1:1 in wt). The FTIR spectra recorded for the two mixtures, reported in Figures S9 and S10, were different from those reported in Figure 2b,c for CuO NPs treated with Cys and Glu, respectively, and corresponded to those obtained for CuO NPs treated with Asp, Leu, Tyr and Phe (where the FTIR of the complexes is superimposed to the spectrum of CuO NPs).

### 3.2. DSC-TGA

Thermal analysis of pristine and treated CuO NPs was carried out in the 30–600 °C temperature range. TG and DSC curves of all the nano-based materials are reported in Figure 3 and, for each material, in Figures S11–S19, while the observed mass losses and DSC peaks are summarized in Table S2.

**Figure 3:**
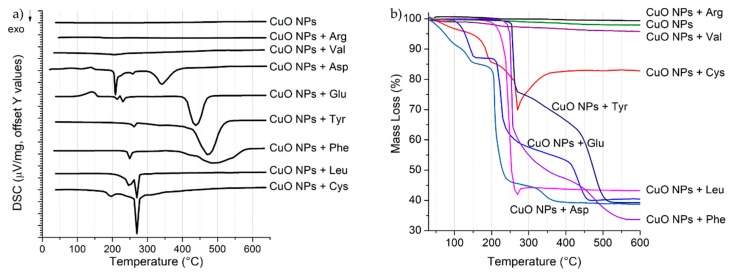
(**a**) Differential Scanning Calorimetry (DSC) and (**b**) Thermo-Gravimetric Analysis (TGA) analysis of CuO NPs and CuO NPs with the different amino acids.

TGA analysis of pristine CuO NPs (Figure S11) did not reveal any significant mass loss (<2.5%) and DSC peaks in the temperature range investigated, indicating that, as already observed by FTIR analysis, only a low amount of organic components and/or water was adsorbed on pristine NPs surface. Similarly, the thermograms of CuO NPs treated with Arg and Val (Figures S12 and S13) showed mass losses of around 0.7% and 4%, respectively. This low decrease, together with the absence of significant DSC peaks (Figure 3a), suggests that these amino acids were not attached on the NPs surface, thus confirming the results obtained by FTIR. The low mass loss observed in TGA curves could be therefore related to few residues of amino acid and /or complex remained on the CuO NPs after washing.

In the case of Cys, the thermogravimetric analysis (Figure S14) showed a first mass loss below 140 °C related to an endothermic DSC peak, probably due to water release. Two other major mass losses, associated to three exothermic reactions, were observed at 140–200 °C and 200–270 °C. Finally, a mass increase was detected in the range 270–360 °C and was associated to an exothermic peak at 310 °C. The observed mass losses can be attributed to a two-step thermal decomposition (dehydration and deamination) and to the decomposition of the residue in different competing pathways (deamination, decarboxylation and pyrolysis of the amino acid residue), as suggested by the presence of the double DSC peak. The mass increase (of about 10%) observed after 270 °C can be ascribed to the oxidation of the Cys thiol group. As already mentioned, Cys is known to bind Cu(II) through the –SH group, which was probably set free to be oxidized under the air flow after the thermal decomposition of the organic part.

A slight increase (<2%) due to oxidation was observed also for CuO NPs treated with Leu (Figure S15), taking place after a two-step decomposition (DSC peaks at 248 and 272 °C) of the organic part between 200 and 270 °C.

As in the previous case, CuO NPs treated with Glu and Asp (Figures S16 and S17) showed a mass loss below 200 °C that can be ascribed to water, followed by a decomposition step in the 200–300 °C range associated to the competing exothermic reactions of decarboxylation and deamination. This was confirmed by the multiple DSC peaks registered. Differently from Cys and Leu, a further mass loss was observed over 300 °C, due to complete decomposition of the organic residue.

Finally, CuO NPs treated with Phe and Tyr (Figures S18 and S19) showed comparable TG and DSC curves, as expected from the similarity between the chemical structures of the two amino acids. A decomposition characterized by two main steps was observed, ascribable to direct decarboxylation and concerted rupturing of C–C bonds with loss of the organic radicals C_7_H_7_-phenyl and, over 400 °C, pyrolysis of the amino acidic residue [16].

DSC and TG analysis confirm the results obtained from FTIR spectra for CuO NPs treated with Arg and Val. However, the differences between the mass loss observed for CuO NPs treated with Cys, Asp, Glu, Leu, Phe and Tyr, as well as the shape of the different curves, do not allow to differentiate between materials where amino acids are bonded to CuO NPs and mixtures of CuO NPs and copper-amino acid complexes.

### 3.3. Total Copper Content in Pristine CuO NPs, Treated CuO NPs and Cu-Amino Acid Complexes

The total content of copper determined in each sample by ICP-MS is reported in Table 2, together with the Cu content estimated for treated CuO NPs by TG analysis. In detail, to compare ICP-MS data with TGA results, the residual weight (%) obtained from TG analysis was converted into Cu (%) by assuming that the residue, at 600 °C in the oxidant gas mixture (33% air), was in the form of CuO. The residual mass of Cys and Leu were determined at 250 °C, before the mass increase ascribed to oxidation processes. A good accordance was found between the values obtained from the two techniques used.

CuO NPs treated with both Val and Arg showed the same percentage of copper than pristine CuO NPs, confirming the formation of soluble copper-complexes which were removed after washing. For NPs treated with Cys, copper content was about 30% lower than for pristine NPs, while for those treated with Asp, Glu, Leu, Phe and Tyr the decrease was around 60–70%.

In the case of Cu-amino acid complexes, the ICP-MS results showed for all the amino acids the formation of Cu(II) complexes with an average stoichiometry of 1:2 (copper:amino acid), as reported in the literature for these complexes [17,18,25,27].

The higher percentage of copper in CuO NPs treated with Cys compared to NPs treated with Glu indicates a different surface coverage that could be due: (i) to the different functional groups by which the two amino acids interact with CuO NPs’ surface; (ii) to the highest solubility of Cys in water with respect to Glu. Depending on the functional groups involved in the bonding, a different number of amino acids could get attached on the NPs’ surface. Regarding the different solubility in water, once Cys is bonded to NPs’ surface and the coverage is completed, the excess of amino acid and/or copper complex is removed from the system during the washing steps with water and EtOH, where both are totally soluble, while insoluble Glu derivatives can remain mixed with the functionalized CuO NPs.

On the other side, Asp, Leu, Phe and Tyr that seem to favor the ligand-assisted ion release from CuO NPs, probably formed poorly water-soluble copper complexes until the free amino acid was totally consumed, leading to final materials containing both CuO NPs and the complexes.

### 3.4. SEM

CuO NPs were analyzed by SEM (Figure S20a). As already reported for these materials [29], CuO NPs present spherical particles with diameters of around 15–20 nm, which resulted highly agglomerated. Similar agglomerates were observed also for CuO NPs treated with Val and Cys (Figure S20b,c). However, in the case of Val, which are likely to dissolve NPs up to the total consumption of the amino acid, SEM images showed mostly bigger agglomerates since they are probably dissolved more slowly than the smaller ones. Finally, CuO NPs treated with Tyr (Figure S20d) showed a material mainly formed by crystals (probably corresponding to Cu-Tyr complexes), while it is not possible to clearly distinguish the presence of nanoparticles.

### 3.5. Colloidal Characterization

The colloidal properties of CuO NPs, both pristine and after interaction with amino acids, were systematically investigated in ultrapure water by means of light scattering techniques. In detail, DLS, ELS and CSA were used to determine hydrodynamic size, zeta potential and sedimentation velocity values of the corresponding water dispersions. The results are reported in Table 3.

### 3.6. DLS

From DLS results, it appears that CuO NPs agglomeration occurred after the addition of all the amino acids investigated, leading to bigger agglomerates with respect to the pristine NPs except for Val and Arg, which showed hydrodynamic size values closer to those of pristine particles. These results confirm, as already observed by FTIR, DSC-TGA and ICP-MS analysis, that Arg and Val formed water-soluble complexes, which are removed from CuO NPs during the washing step.

An increase of the hydrodynamic size up to 4 μm was observed for the CuO NPs treated with the other amino acids, indicating that both the surface coating and the presence of insoluble copper complexes are influencing the measurement.

### 3.7. ELS

The influence of amino acids on CuO NPs zeta potential values was investigated by means of ELS technique (Table 3). In detail, amino acids forming water soluble complexes (i.e., Val and Arg) led to NPs showing positive zeta potential values close to those of pristine CuO NPs, but slightly reduced. Zeta potential values decreased from about 15 mV for pristine to 13 and 8 mV for CuO NPs treated with Val and Arg, respectively. These variations can be due to the dissolution process occurring to CuO NPs in the presence of these amino acids.

In the case of amino acids with a negatively charged side chain such as Asp and Glu, which despite the similar chemical structures showed a different kind of interaction with CuO NPs, zeta potentials values were around the point of zero charge for both amino acids but with opposite charge (−0.3 and 1.3 mV respectively). Negative values of zeta potential were measured for CuO NPs treated with Cys, as well as with the remaining amino acids, all bearing hydrophobic side chains. However, while in the case of Cys and Glu the results can be attributed to the presence of an amino acidic coating, for the other amino acids the presence of the insoluble complexes in the dispersion media was probably influencing these values.

### 3.8. CSA

To conclude the colloidal characterization, sedimentation velocity of the different dispersions was measured by CSA. Very low values were obtained for pristine CuO NPs and for those treated with Val and Arg (ranging from 0.03 to 0.07 μm/s), indicating a similar and quite good colloidal stability for all the samples. CuO NPs treated with all the other amino acids showed up to 10 times higher sedimentation velocity values (around 0.3 μm/s). In general, high sedimentation velocity values correspond to high colloidal instability of the analyzed dispersion. These results are in accordance with the hydrodynamic size values observed by DLS.

### 3.9. Antibacterial Test

**Figure 4:**
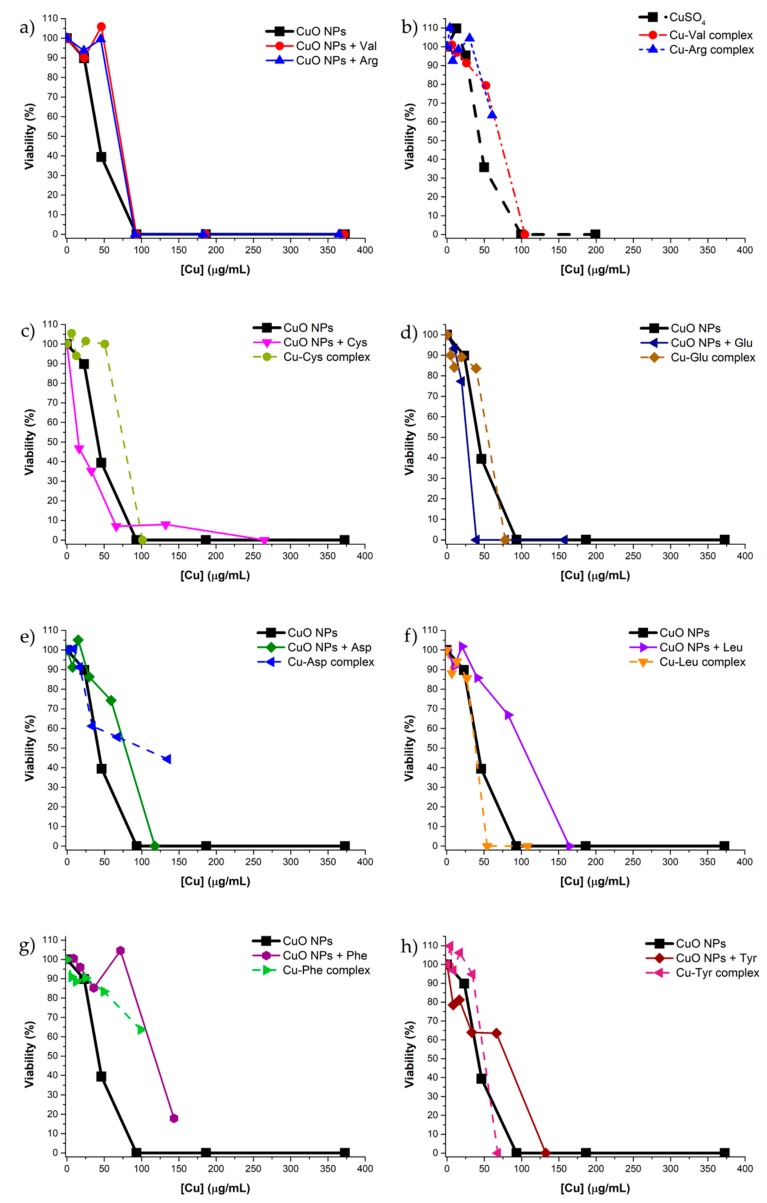
Viability (%) of the Gram-positive bacterium *Staphylococcus epidermidis* ATCC 35984 vs. copper concentration in (**a**) pristine CuO NPs, CuO NPs treated with Val and CuO NPs treated Arg; (**b**) CuSO4, Cu-Val complex and Cu-Arg complex; (**c**) pristine CuO NPs, CuO NPs treated with Cys and Cu-Cys complex; (**d**) pristine CuO NPs, CuO NPs treated with Glu and Cu-Glu complex; (**e**) pristine CuO NPs, CuO NPs treated with Asp and Cu-Asp complex; (**f**) pristine CuO NPs, CuO NPs treated with Leu and Cu-Leu complex; (**g**) pristine CuO NPs, CuO NPs treated with Phe and Cu-Phe complex and (**h**) pristine CuO NPs, CuO NPs treated with Tyr and Cu-Try complex.

The antibacterial activity of CuO NPs (both pristine and treated with amino acids), Cu-amino acid complexes and CuSO_4_ was tested against the Gram-positive bacterium *Staphylococcus epidermidis* ATCC 35984, a model microorganism for nosocomial infections and well-known biofilm forming pathogen. The results are reported in Figure 4 as % viability vs. copper concentration, instead of material concentration, in order to compare the toxicity of the different materials as a function of copper content (as previously determined by ICP-MS). MIC values for each tested material are reported in Table 4. Free amino acids were also tested as controls, showing no antimicrobial activity at the concentration of 500 μg/mL.

As expected, CuO NPs treated with Val and Arg (Figure 4a) showed a similar antimicrobial activity than pristine NPs, with no significant differences in the calculated MIC (around 90 μg/mL). A comparable antimicrobial activity was observed for the corresponding complexes, as well as for CuSO_4_ (Figure 4b). These results suggested that the release of copper ions was the main factor responsible for the toxic effect on *S. epidermidis*, in accordance with the findings of Bondarenko et al. [30].

The CuO NPs treated with Cys (Figure 4c) seem to be more active than pristine NPs at concentrations up to 70 μg/mL; however, this material allowed to reach the total mortality of bacteria only at 265 μg/mL. The observed effect could be due to the Cys coating present on the NPs’ surface. The corresponding Cu-Cys complex presented a similar toxicity than pristine CuO NPs, resulting more active than the functionalized CuO NPs in terms of MIC values.

CuO NPs treated with Asp and Glu, despite the comparable copper content and the similar chemical structure of the two amino acids, presented a different antibacterial behavior, ascribable to the different way by which they interact with the CuO NPs. As shown in Figure 4d, CuO NPs with Glu exhibited a MIC of 39 μg/mL while in the case of Asp (Figure 4e), the toxicity of the material was lower than that of pristine CuO NPs and the desired antibacterial effect was reached only at 120 μg/mL. These results are probably related to the different bioavailability of copper present in the two materials (i.e., nanoparticles functionalized with Glu and the mixture CuO NPs/Cu-Asp complex). As far as the Cu-Glu and Cu-Asp complexes are concerned, the first one is quite more toxic (MIC 78 vs. >135 μg/mL), suggesting a possible role in the antibacterial activity of the Glu bonded on the NPs’ surface, which is coordinated to copper in a different way with respect to the corresponding complex, as shown by the FTIR analysis.

Comparing Cys and Glu, the only two amino acids that get bonded to the CuO NPs surface, a similar behavior in the first part of the viability curve can be observed (Figure 4c,d), then, at the highest concentrations, Cys showed a reduced toxicity with respect to Glu. This effect could be due to the different protection of NPs surface performed by the two amino acids against copper dissolution. Moreover, the different surface functionalization could influence the interactions of NPs with the bacteria cell-wall and, consequently, the uptake by *S. epidermidis* [31].

A decrease of the antibacterial activity, compared to the pristine CuO NPs, was observed for the materials containing amino acids with hydrophobic or aromatic side chains, such as Leu, Phe and Tyr (Figure 4f–h). In particular, in the case of CuO NPs treated with Phe, bacteria still exhibited a viability of around 20% at the highest copper concentration tested (MIC > 143 μg/mL). In contrast, while the Cu-Phe complex showed a lower antibacterial activity than pristine CuO NPs (MIC > 99 μg/mL), Cu-Leu and Cu-Tyr complexes resulted more toxic (MIC of 54 and 68 μg/mL, respectively). Although CuO NPs treated with these amino acids were expected to exhibit an intermediate antibacterial behavior between pristine CuO NPs and the corresponding complexes, it must be considered that the combination of surface charge, side chain hydrophobicity, the presence of copper-amino acid complexes and their interactions with the NPs’ surface could limit the availability of copper in these materials.

## 4. Conclusions

The use of multiple analytical techniques allowed to highlight how the interactions between CuO NPs and natural amino acids may be strongly influenced by the nature of the aminoacidic side chain and how, in turn, they may affect the antimicrobial activity of CuO NPs. In detail, Arg and Val lead to the formation of Cu(II) water soluble complexes and no aminoacidic residues were found on the NPs’ surface. Conversely, in accordance with the literature, the linkage of Cys and Glu on CuO NPs surface was observed by FTIR. Finally, the formation of poorly soluble Cu(II) complexes was detected following the treatment of CuO NPs with Asp, Leu, Phe and Tyr. The antimicrobial activity of CuO NPs against the Gram-positive bacterium *S. epidermidis* ATCC 35984 was highly influenced by the presence of amino acids. With respect to pristine CuO NPs, the results ranged from a higher antibacterial activity shown by Glu-treated CuO NPs (where Glu is supposed to be bonded on CuO NPs’ surface) and the lower effects exerted by CuNPs treated with Asp, Leu, Phe and Tyr (which are physical mixtures of CuO NPs with poorly water-soluble Cu–amino acid complexes). A particular behavior, which could be associated to the extent of NP surface coating, was observed as a result of the interaction of CuO NPs with Cys. The differences observed for CuO NPs functionalized with Cys and Glu could be ascribed to both the different protection exerted by these amino acids against NPs’ surface dissolution and the different way by which the functionalization may influence the interaction of these materials with bacteria. Finally, the presence of both insoluble complexes and CuO NPs as a physical mixture always led to less toxic materials with respect to the pristine, even when the corresponding complexes were highly toxic for the bacteria investigated, indicating a possible masking effect between NPs and the complexes.

The results of this study may contribute to the understanding of the interactions between CuO NPs and amino acids, which are highly abundant in living systems. The knowledge acquired on these nano-bio interactions could be also helpful for the purpose of safer product design, and improved product performance, especially in the field of cultural heritage conservation.

## Figures and Tables

**Table 1:** Inductively Coupled Plasma Mass Spectrometry (ICP-MS) Parameters.

Component/Parameter	Type/Value/Mode
Nebulizer	Meinhard quartz microconcentric
Spray Chamber	Quartz cyclonic
Triple Cone Interface Material	Nickel/Aluminum
Plasma Gas Flow	18 L/min
Auxiliary Gas Flow	1.2 L/min
Nebulizer Gas Flow	0.96–1 L/min
Sample Uptake Rate	200–250 µL/min
RF Power	1600 W
Isotope	^63^Cu

**Table 2:** Total copper content in pristine and treated CuO NPs measured by Inductively Coupled Plasma Mass Spectrometry (ICP-MS) and calculated on the basis of TG analysis, and total copper content in Cu-amino acid complexes measured by ICP-MS.

	NPs		Complexes
	ICP-MS Cu (%)	TGA Cu (%)	ICP-MS Cu (%)
CuO	74.6 ± 0.6	78	-
Val	74.4 ± 0.5	77	20.9 ± 0.5
Arg	73.0 ± 0.3	80	12.1 ± 0.7
Cys	53.0 ± 0.3	55	20.3 ± 0.6
Asp	23.5 ± 0.3	29	27.0 ± 0.6
Glu	31.5 ± 0.5	32	15.6 ± 0.5
Leu	32.9 ± 0.4	30	21.7 ± 1.6
Phe	28.7 ± 0.2	27	19.8 ± 0.5
Tyr	26.5 ± 0.7	31	13.6 ± 0.7

**Table 3:** Hydrodynamic size, zeta potential (ξ) and sedimentation velocity values of pristine CuO NPs and treated with amino acids, dispersed in ultrapure water.

NPs	Hydrodynamic Size (nm)	ξ (mV)	Sedimentation Velocity (μm/s)
CuO	340 ± 206	15.4 ± 0.6	0.03 ± 0.01
CuO + Val	839 ± 182	13.1 ± 1.0	0.04 ± 0.01
CuO + Arg	518 ± 103	7.8 ± 0.9	0.07 ± 0.01
CuO + Cys	2630 ± 1031	−4.3 ± 0.8	0.26 ± 0.01
CuO + Asp	2714 ± 1371	−0.3 ± 0.7	0.33 ± 0.01
CuO + Glu	2084 ± 1053	1.3 ± 1.7	0.22 ± 0.03
CuO + Leu	2803 ± 1522	−8.1 ± 1.9	0.34 ± 0.03
CuO + Phe	1491 ± 895	−4.0 ± 0.5	0.22 ± 0.01
CuO + Tyr	4205 ± 1009	−6.5 ± 1.4	0.28 ± 0.01

**Table 4:** Minimal Inhibitory Concentration (MIC) values of the different materials tested. All the concentrations refer to Cu content measured by ICP-MS (see Table 2).

Materials	MIC (μg Cu/mL)	Materials	MIC (μg Cu/mL)
CuO NPs	94	CuSO_4_	99
CuO NPs + Val	93	Cu-Val complex	104
CuO NPs + Arg	91	Cu-Arg complex	>60
CuO NPs + Cys	265	Cu-Cys complex	101
CuO NPs + Asp	120	Cu-Asp complex	>135
CuO NPs + Glu	39	Cu-Glu complex	78
CuO NPs + Leu	165	Cu-Leu complex	54
CuO NPs + Phe	>143	Cu-Phe complex	>99
CuO NPs + Tyr	135	Cu-Tyr complex	68

## Supplementary Materials

The following are available online at https://www.mdpi.com/2079-4991/9/5/792/s1, Figure S1: Attenuated Total Reflection (ATR)-FTIR spectra of Val, Cu-Val complex and CuO NPs treated with Val, Figure S2: ATR-FTIR spectra of Arg, Cu-Arg complex and CuO NPs treated with Arg, Figure S3: ATR-FTIR spectra of Cys, Cu-Cys complex and CuO NPs treated with Cys, Figure S4: ATR-FTIR spectra of Glu, Cu-Glu complex and CuO NPs treated with Glu, Figure S5: ATR-FTIR spectra of Asp, Cu-Asp complex and CuO NPs treated with Asp, Figure S6: ATR-FTIR spectra of Leu, Cu-Leu complex and CuO NPs treated with Leu, Figure S7: ATR-FTIR spectra of Phe, Cu-Phe complex and CuO NPs treated with Phe, Figure S8: ATR-FTIR spectra of Tyr, Cu-Tyr complex and CuO NPs treated with Tyr, Figure S9: ATR-FTIR spectra of CuO NPs/Cu-Cys complex physical mixture, Figure S10: ATR-FTIR spectra of CuO NPs/Cu-Glu complex physical mixture, Figure S11: DSC and TGA spectra of pristine CuO NPs, Figure S12: DSC and TGA spectra of CuO NPs treated with Arg, Figure S13: DSC and TGA spectra of CuO NPs treated with Val, Figure S14: DSC and TGA spectra of CuO NPs treated with Cys, Figure S15: DSC and TGA spectra of CuO NPs treated with Leu, Figure S16: DSC and TGA spectra of CuO NPs treated with Glu, Figure S17: DSC and TGA spectra of CuO NPs treated with Asp, Figure S18: DSC and TGA spectra of CuO NPs treated with Phe, Figure S19: DSC and TGA spectra of CuO NPs treated with Tyr, Figure S20. SEM images of (a) pristine CuO NPs; (b) CuO NPs treated with Val; (c) CuO NPs treated with Cys; (d) CuO NPs treated with Tyr, Table S1: Infrared band assignment of free amino acids (Leu, Glu, Asp, Tyr, Phe, Cys) and CuO NPs treated with Leu, Glu, Asp, Tyr, Phe and Cys, according to the literature, Table S2: TG and DSC analysis results.
