# Supplementary material for: Interaction between Copper Oxide Nanoparticles and Amino Acids: Influence on the Antibacterial Activity
Source: Nanomaterials (Basel). 2019 May 23;9(5):792. doi: 10.3390/nano9050792 (PMC6566567; doi:10.3390/nano9050792)
Supplement: Supplementary file 1 [file nanomaterials-09-00792-s001.pdf]

Supplementary Materials

Interaction between Copper Oxide Nanoparticles and Amino Acids: Influence on the Antibacterial Activity

Elena Badetti [1,*], Loris Calgaro [1], Laura Falchi [1], Alessandro Bonetto [1], Cinzia Bettiol [1], Benedetta Leonetti [2,3], Emmanuele Ambrosi [2,3], Elisabetta Zendri [1] and Antonio Marcomini [1,*]

1. DAIS—Department of Environmental Sciences, Informatics and Statistics, University Ca' Foscari of Venice, Via Torino 155, 30172 Venice Mestre, Italy; loris.calgaro@unive.it (L.C.); laura.falchi@unive.it (L.F.); alessandro.bonetto@unive.it (A.B.); bettiol@unive.it (C.B.); elizen@unive.it (E.Z.)
2. DMSN—Department of Molecular Sciences and Nanosystems, University Ca' Foscari of Venice, Via Torino 155/b, 30172 Venice Mestre, Italy; leonetti393@gmail.com (B.L.); emmanuele.kizito@gmail.com (E.A.)
3. ECLT Lab—European Centre for Living Technology, University Ca' Foscari of Venice, Via Torino 155/b, 30172 Venice Mestre, Italy

* Correspondence: elena.badetti@unive.it (E.B.); marcom@unive.it (A.M.)

ATR-FTIR

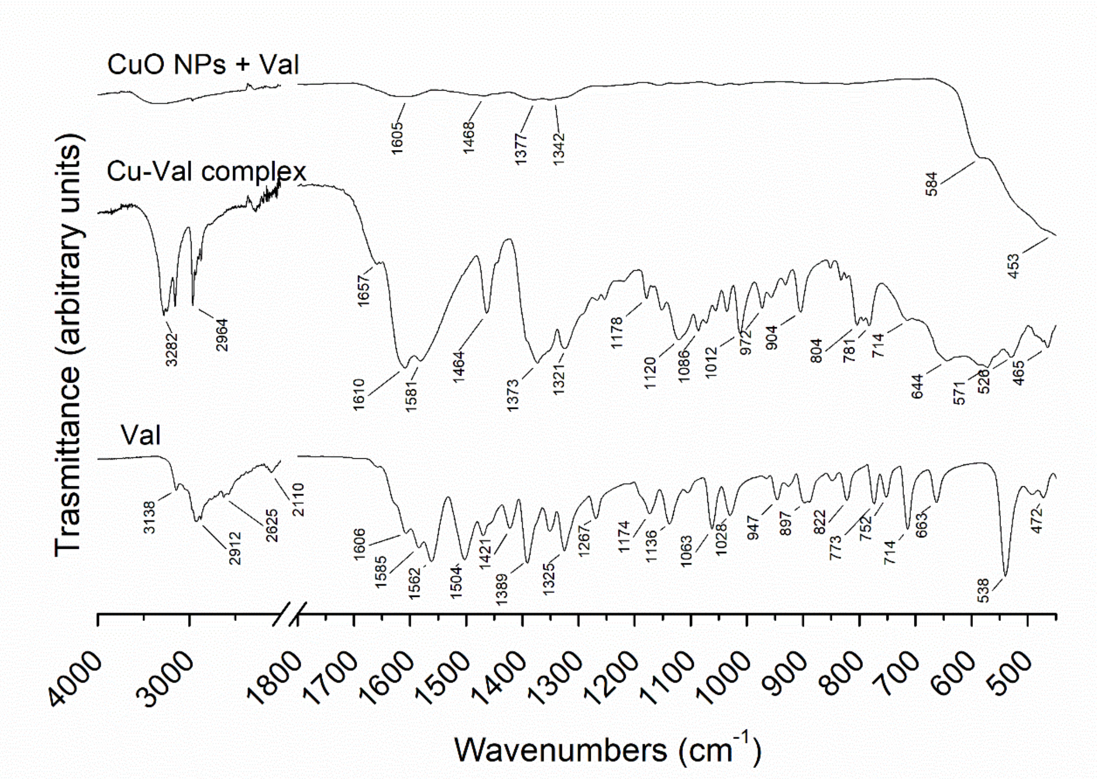

Figure S1. ATR-FTIR spectra of Val, Cu-Val complex and CuO NPs treated with Val.

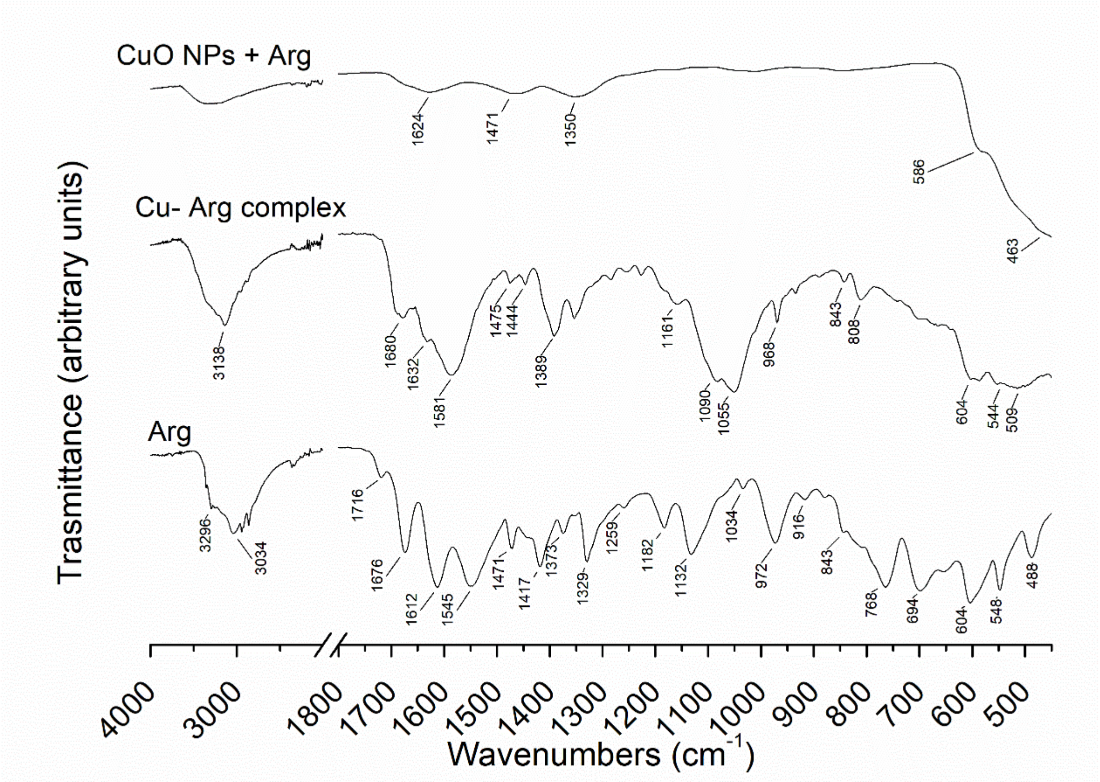

Figure S2. ATR-FTIR spectra of Arg, Cu-Arg complex and CuO NPs treated with Arg.

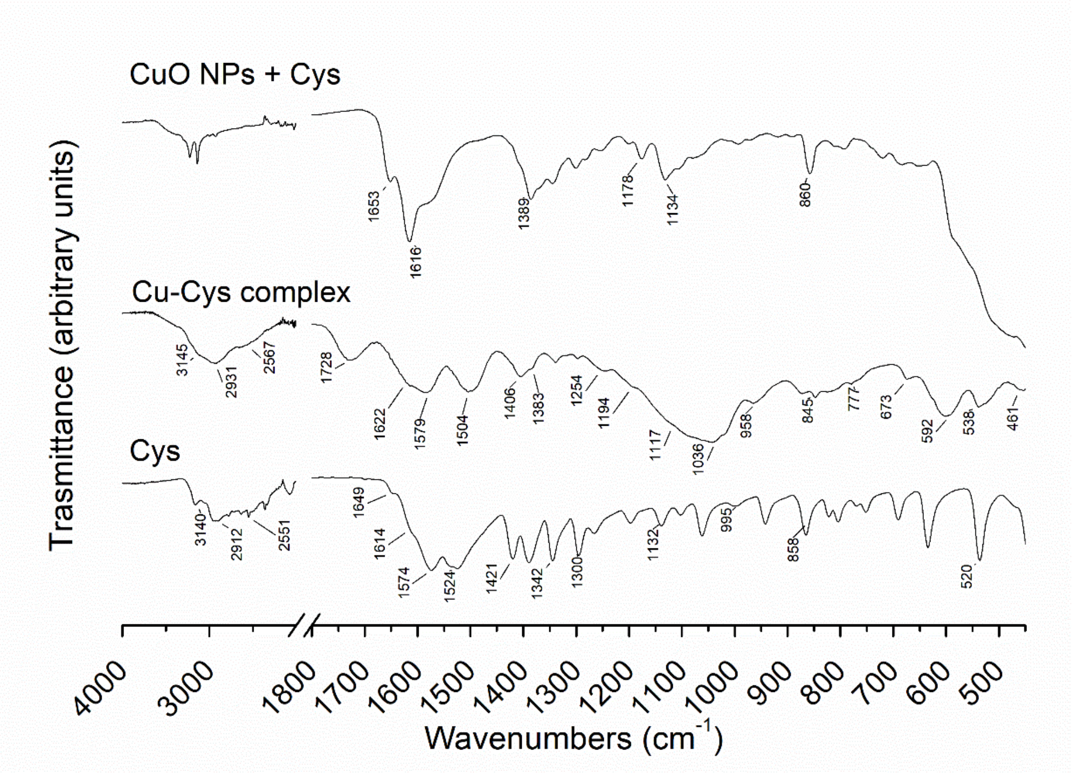

Figure S3. ATR-FTIR spectra of Cys, Cu-Cys complex and CuO NPs treated with Cys.

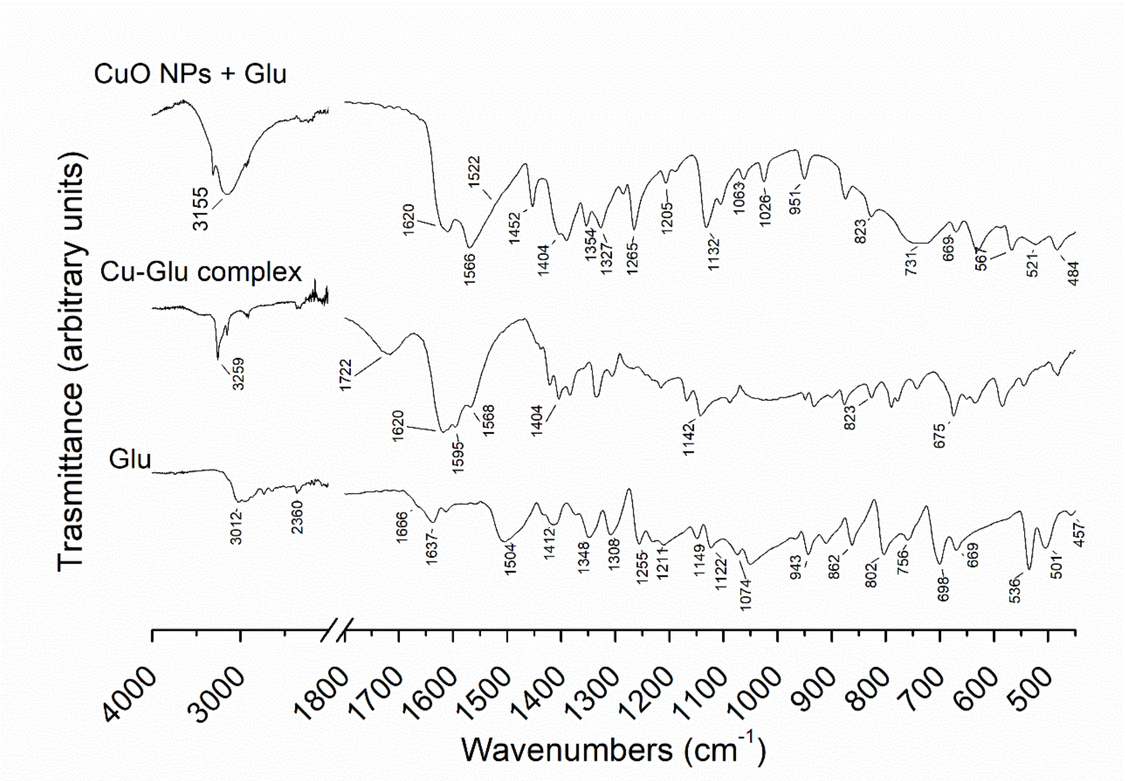

Figure S4. ATR-FTIR spectra of Glu, Cu-Glu complex and CuO NPs treated with Glu.

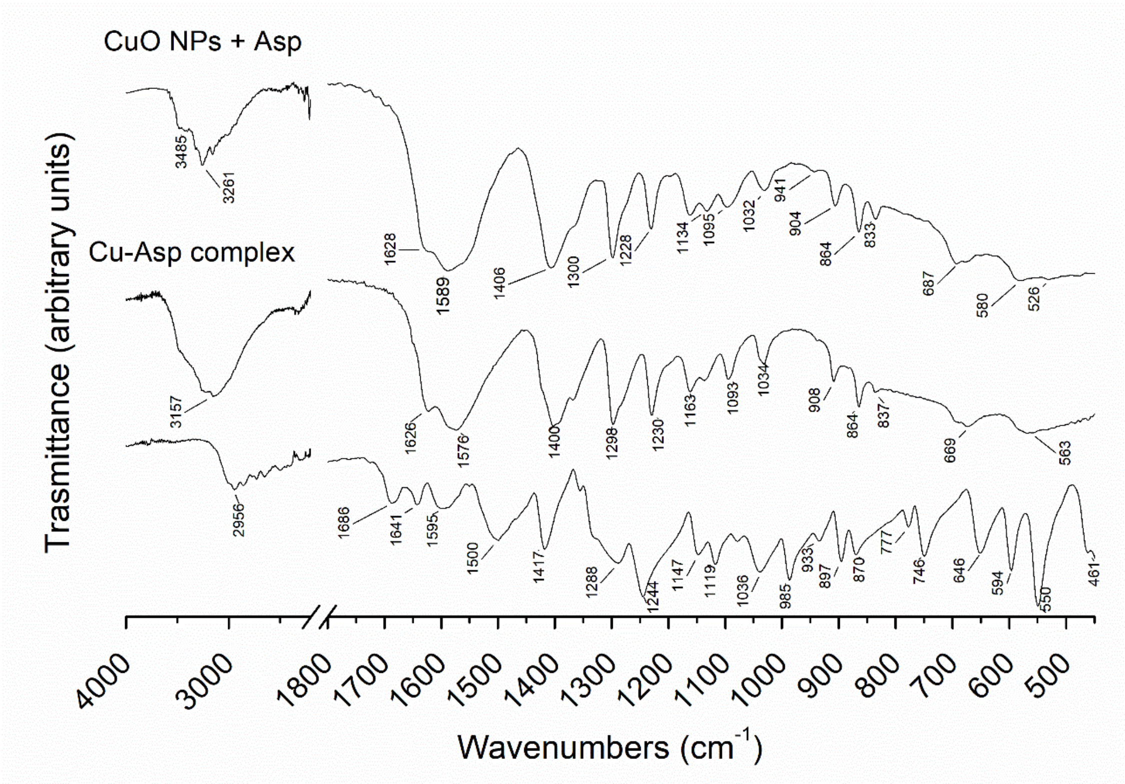

Figure S5. ATR-FTIR spectra of Asp, Cu-Asp complex and CuO NPs treated with Asp.

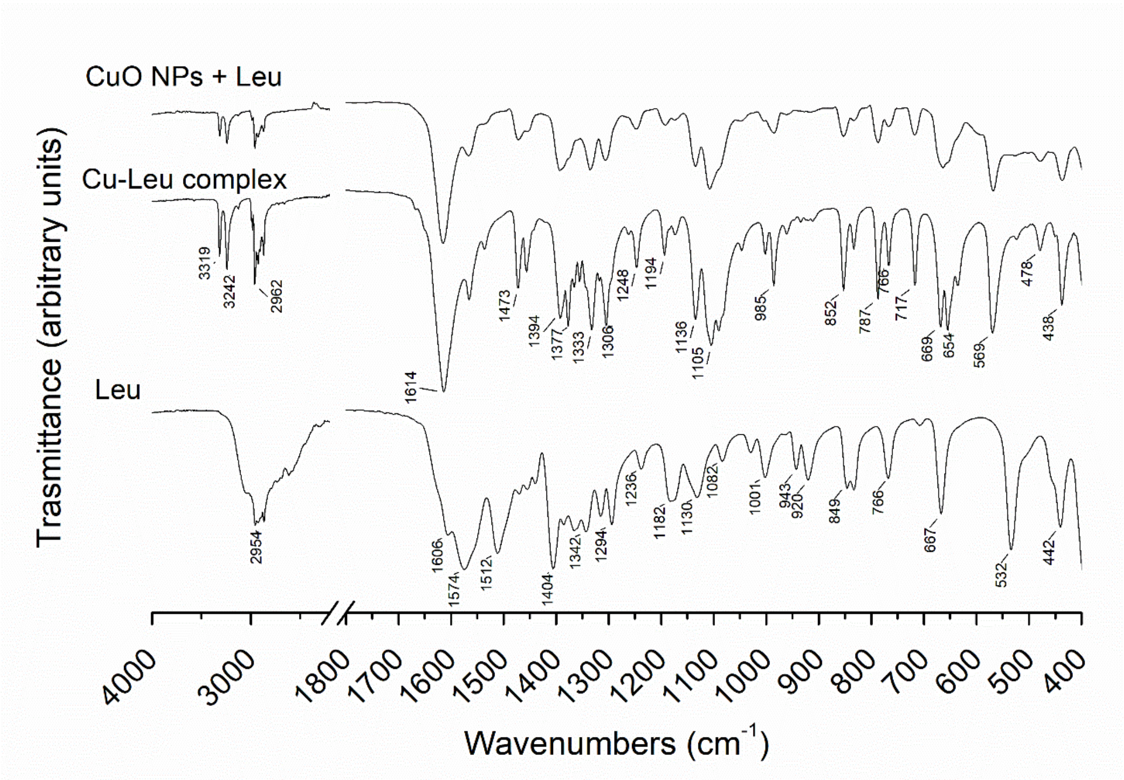

Figure S6. ATR-FTIR spectra of Leu, Cu-Leu complex and CuO NPs treated with Leu.

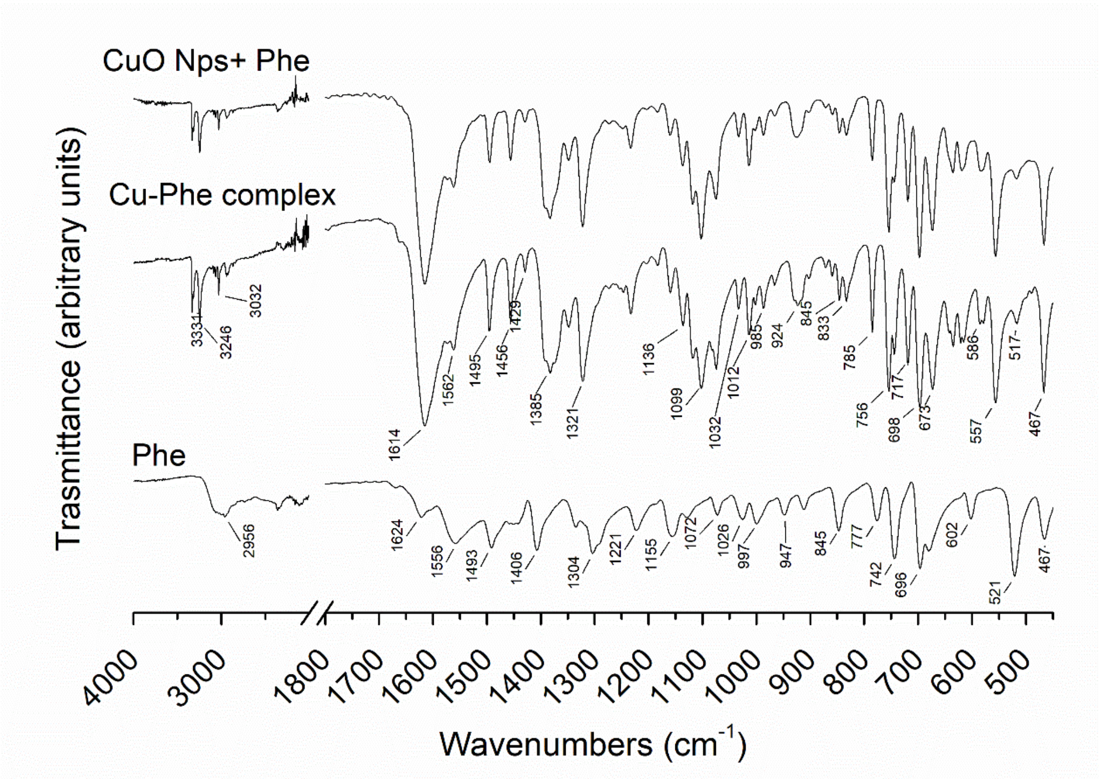

Figure S7. ATR-FTIR spectra of Phe, Cu-Phe complex and CuO NPs treated with Phe.

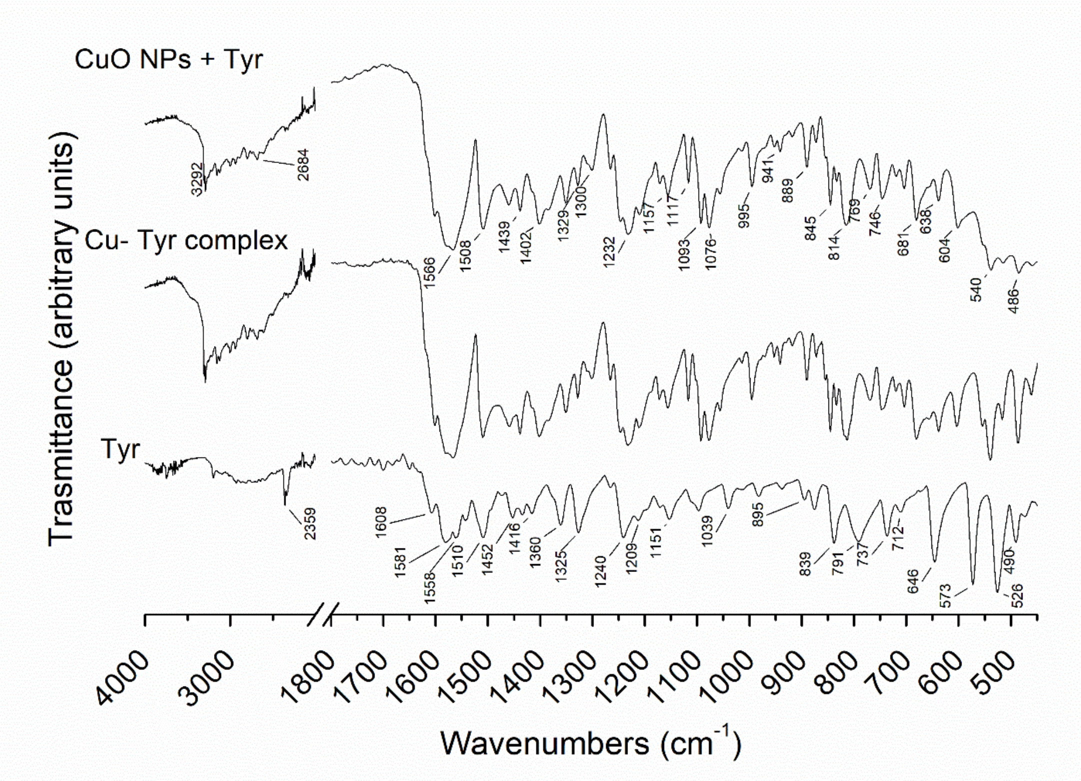

Figure S8. ATR-FTIR spectra of Tyr, Cu-Tyr complex and CuO NPs treated with Tyr.

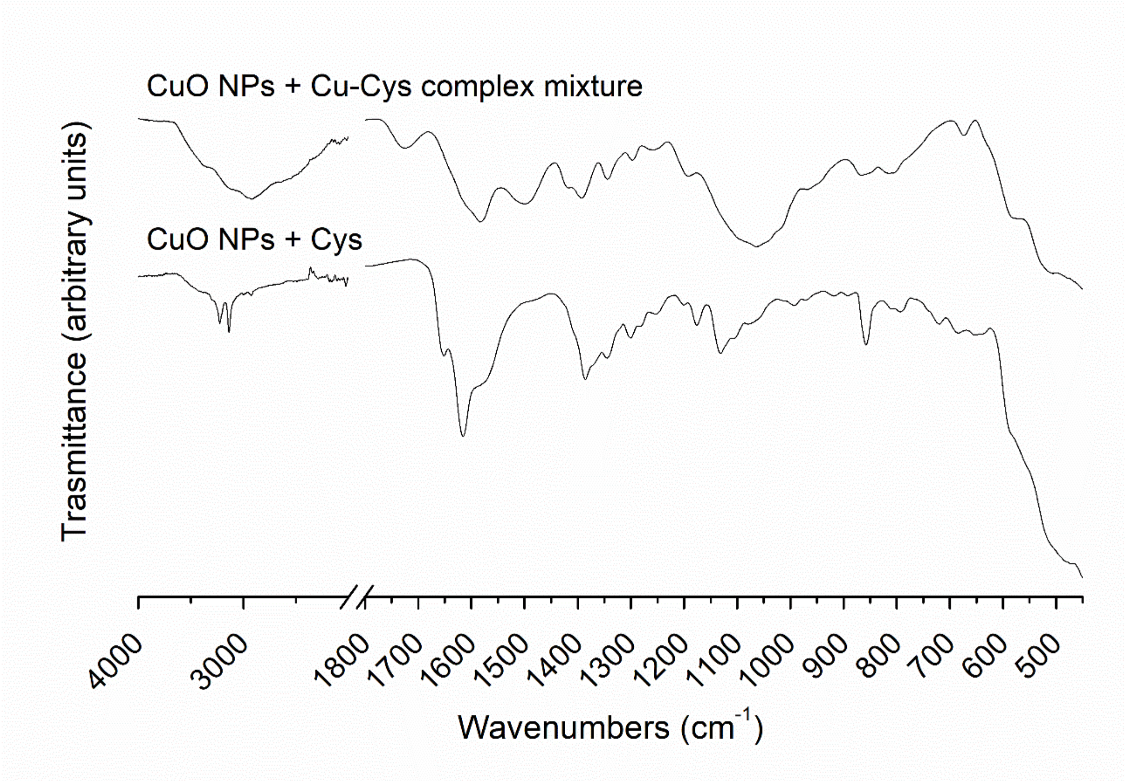

Figure S9. ATR-FTIR spectra of CuO NPs/Cu-Cys complex physical mixture.

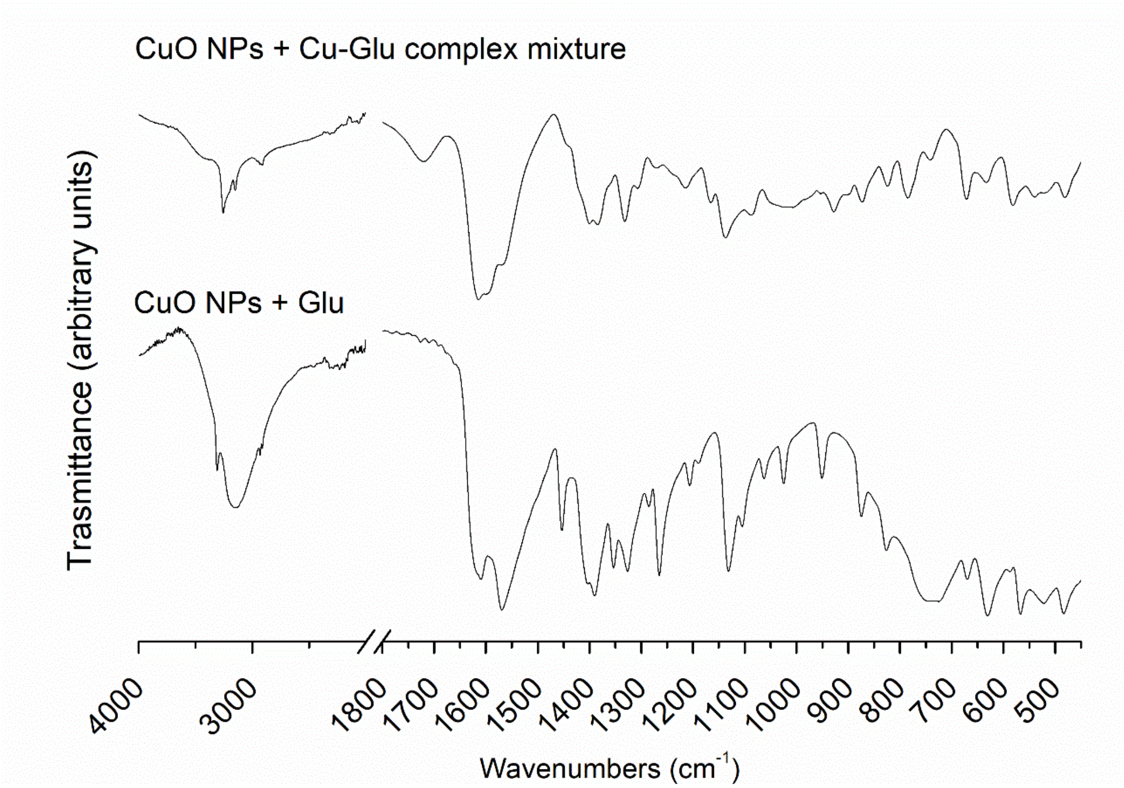

Figure S10. ATR-FTIR spectra of CuO NPs/Cu-Glu complex physical mixture.

Table S1. Infrared band assignment (cm^{-1}) of free amino acids (Leu, Glu, Asp, Tyr, Phe, Cys) and CuO NPs treated with Leu, Glu, Asp, Tyr, Phe and Cys, according to the literature [1–6].

**Assignment** [1]	**Leu**	**CuO NPs+Leu**	**Glu**	**CuO NPs+Glu**	**Asp**	**CuO NPs+Asp**	**Tyr**	**CuO NPs+Tyr**	**Phe**	**CuO NPs+Phe**	**Cys**	**CuO NPs+Cys**
ν CH phenyl-							3202	3307 w	3068 m	3332 m		
ν OH (H_2O)		3315, 3242 s	3044 w	3312 s		3261					3165 s	
ν NH_2; (ν CH phenyl- for Tyr and Phe)	~3000 b		3012 b	3155 b	3017 b	3128 b	3000 b	3157 b	2960 b	3032 b,w		
ν CH_2, CH_3 sharp); ν N–H (broad)	2956, 2868 s	2958 m, 2925 m, 2870 m	2914 b	2928,2910 m	2946,2855 s	2977,2931 m	2922 w	2995,2931 m	2953 b w	2938 b w	2990 s	3226, 3138
ν SH											2551 s	
Overtones, combination bands	2617 w		2736, 2628 w		2727, 2652, 2502 w						2058 b	
ν C=O			1666 w		1686 m				1672		1649 vw	1653
δ_{Asym} N-H	1606 w	1614 vs	1637 m	1612 vs	1641 m	1628 vs	1606 m	1601m	1622 w	1616 s	1614 w	1614 s
ν CC, δ CH; ν_{asym} as COO^- carboxylate	1574 s	1566 w	1614 b	1566vs	1595 m	1589 vs	1576-1559 vs	1567 vs	1558 s	1561 w	1568 s	1574 m b
δ_{sym} N–H; (ν CC and δ CH phenyl for Tyr and Phe)	1512 s	1473 m	1504 s		1500 s	1507 s	1510 s	1493 s1496 m, sh	1512 s	1473 m	1524 s	
δ CH; ν_{sym} COO^-	1454 m, 1406 s	1456 m, b	1435 b, 1412 m	1407-1390 vs, b	1417 s	1406 vs	1456,1418 m	1460-1402 m	1408 s	1456 m, sharp	1421 s,	

**Assignment** [1]	**Leu**	**CuO NPs+Leu**	**Glu**	**CuO NPs+Glu**	**Asp**	**CuO NPs+Asp**	**Tyr**	**CuO NPs+Tyr**	**Phe**	**CuO NPs+Phe**	**Cys**	**CuO NPs+Cys**
δ CH_2, CH_3; δHCN	1385 m	1399 m	1348 s	1354 s	1356m	1367 m	1362 s	1351 m	1336 m	1383 s	1389 s	1385 s
δ COH; (δ CH phenyl for Tyr and Phe)	1342 m,w	1335 m	1308 s	1327 s	1288 s	1300 s	1328 s	1328 w	1304 s	1323 s	1344 s	1342 m
γ CH_2; δ NH; ν CO	1238 m, 1294 s	1246 w	1255	1265 s	1244 m	1228 s	1242 s	1232 s	1223 s	1232 m	1296s, 1265w	1300w, 1277, 1250 vw
δ CH_3			1211 m	1205 w			1213 w	1211 w				
ν (CC)R ; δ (HNC)	1182 m	1194 w	1149 w	1132m	1147 w	1159 m	1174 m	1172	1155 s	1160 w		1174 w
ν CO, δ OH											1196 m	
δ CH; NH_3^+	1132 m	1136 s	1122 m	1105	1119 m	1134 w	1153 m	1156 m	1129 w	1137 m	1140 m	1132 m
δ (HCC)R	1084 w	1106, 1091	1074 w	1063 w	1080 vw	1095 m	1098 m	1093 m	1074 m	1101 s	1101 w	
ν (CC)R; ν (N–C)	1030 vw		1047 s	1026 w	1036 m	1032 m	1041 m	1078 m	1024 m	1074 m	1063 s	1078 w
δ SH											995 m	993 vw
ν (CC)	1001m	1007+986 m	943 s	951 m	985 m	941vw	983 w	995 m	1001 m	1014 m	943 s	
γ (HCCH)R	943m		908 m		933 w	904 w	895	890m	912	924		
ν COO^{-};	847 m	852m	862	876m	897 m	864 w	839 s	845w	849 s	846 w	866 s	858 m
δ HNH	831 w	833 m	802 s	823	870		813				822, 804 w	
γ CC	768m	791 m	756 w		746 s	833 w	792 m	769 w	773s	785 m	770 vw	789 w
γ COO^- scissor; δ CH out of plane def		767 m	698 s	731 s		687 m	738	747 w	744 vs	754 s	752 w	752 vw

Assignment [1]	Leu	CuO NPs+Leu	Glu	CuO NPs+Glu	Asp	CuO NPs+Asp	Tyr	CuO NPs+Tyr	Phe	CuO NPs+Phe	Cys	CuO NPs+Cys
Out of plane phenyl def for Tyr and Phe							712 w	704 w	697 vs	698 s		
ν CS											690 m,s	683 vw
ν CS, γ COO^-											636 s	
γ COO^- ; NH_2 rock (complexes)	667s	669-665 m	669 w	669 w	646 s	672 w	669 w	681 w681 m	674 m			
In plane phenyl def							574 vs	602 m	604 m	582 w		
γ COO^- ; γ COO^-N−H	532s	567	5536 w, 501 m	484 m	594, 550	580	523 vs	538 m	521m	555 s	536 s	
ν CuO		478 w, b		450 m, b		526 s, b		485 m		467vw		499 s

b=broad; s=strong, m=medium, w=week; sh=sharp; v=very

DSC-TGA

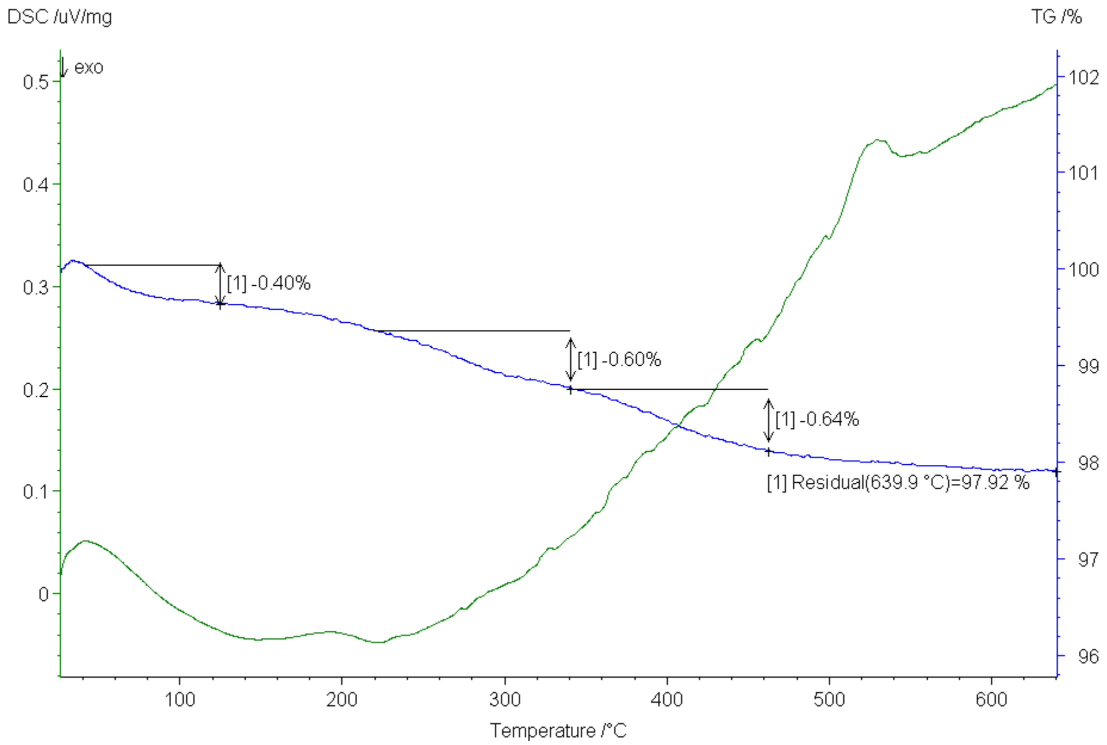

Figure S11. DSC and TGA spectra of of pristine CuO NPs.

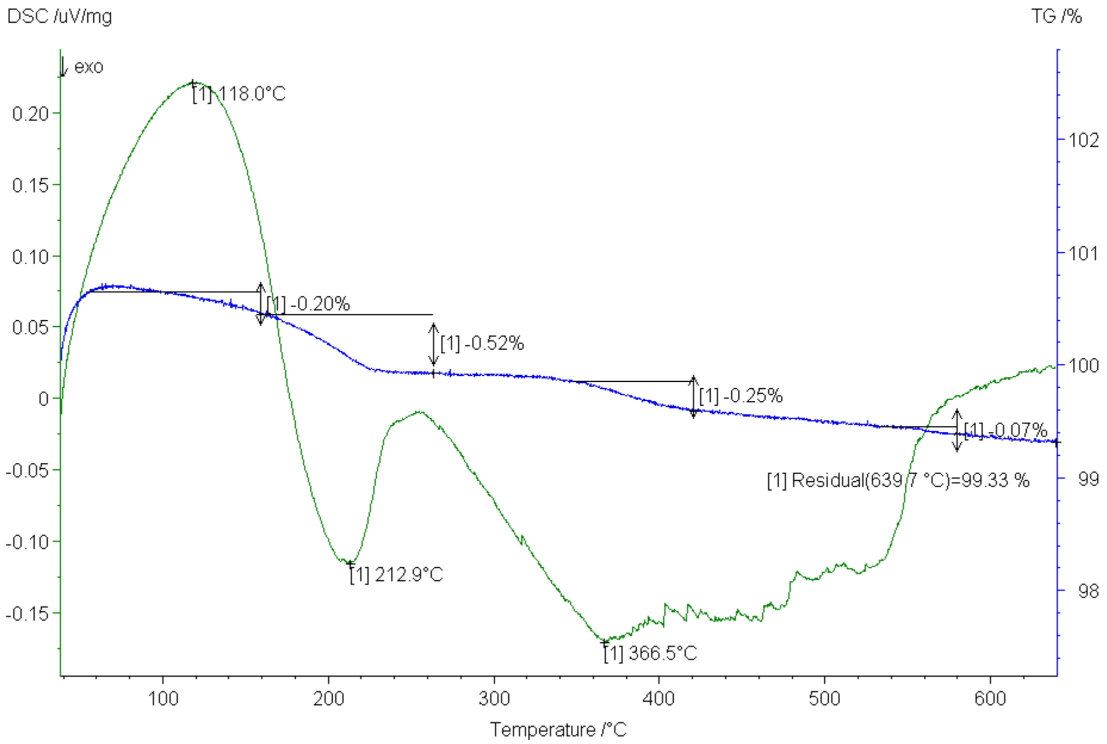

Figure S12. DSC and TGA spectra of CuO NPs treated with Arg.

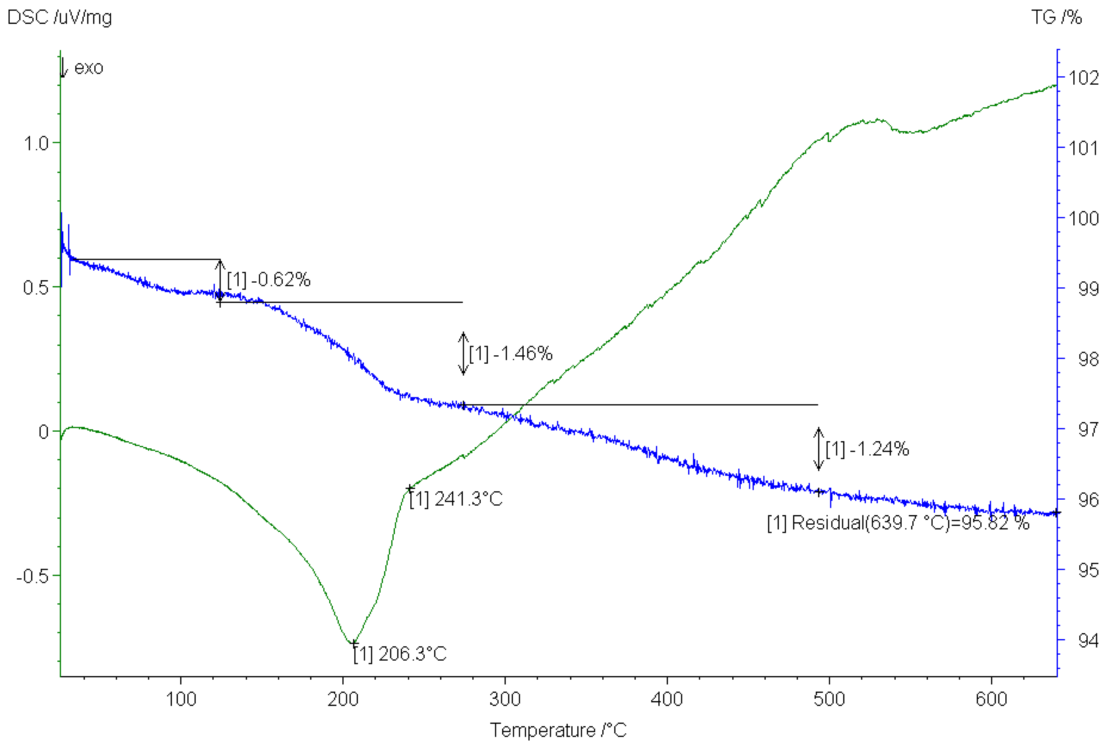

Figure S13. DSC and TGA spectra of CuO NPs treated with Val.

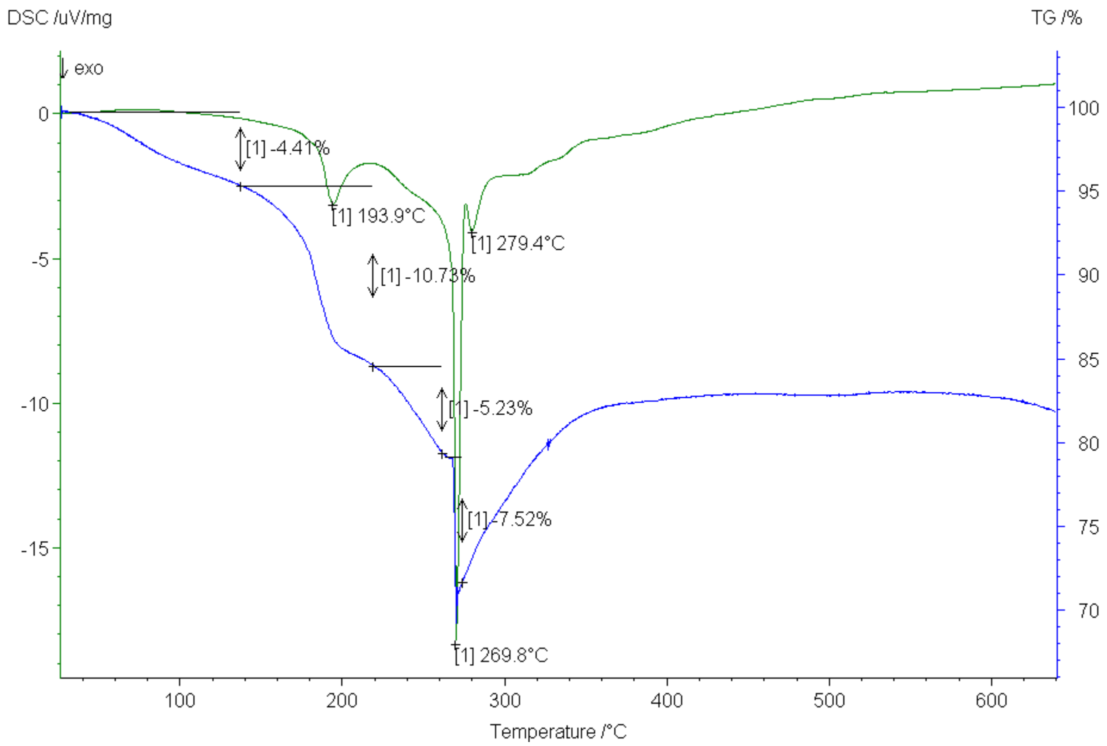

Figure S14. DSC and TGA spectra of CuO NPs treated with Cys.

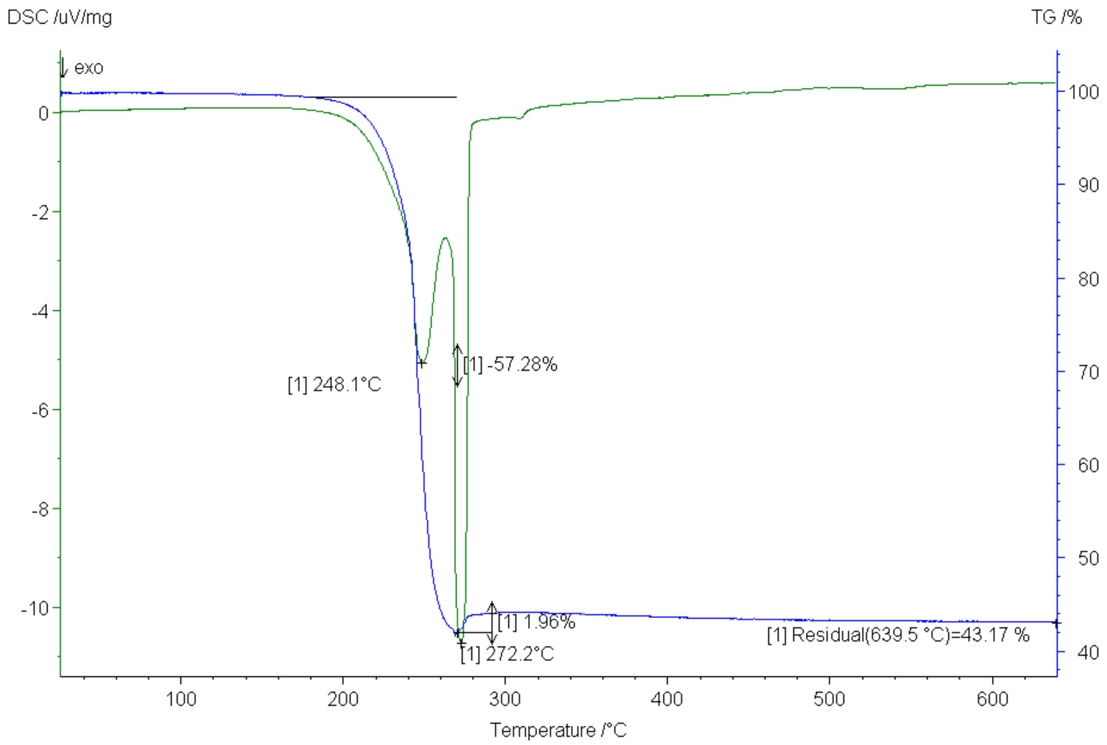

Figure S15. DSC and TGA spectra of CuO NPs treated with Leu.

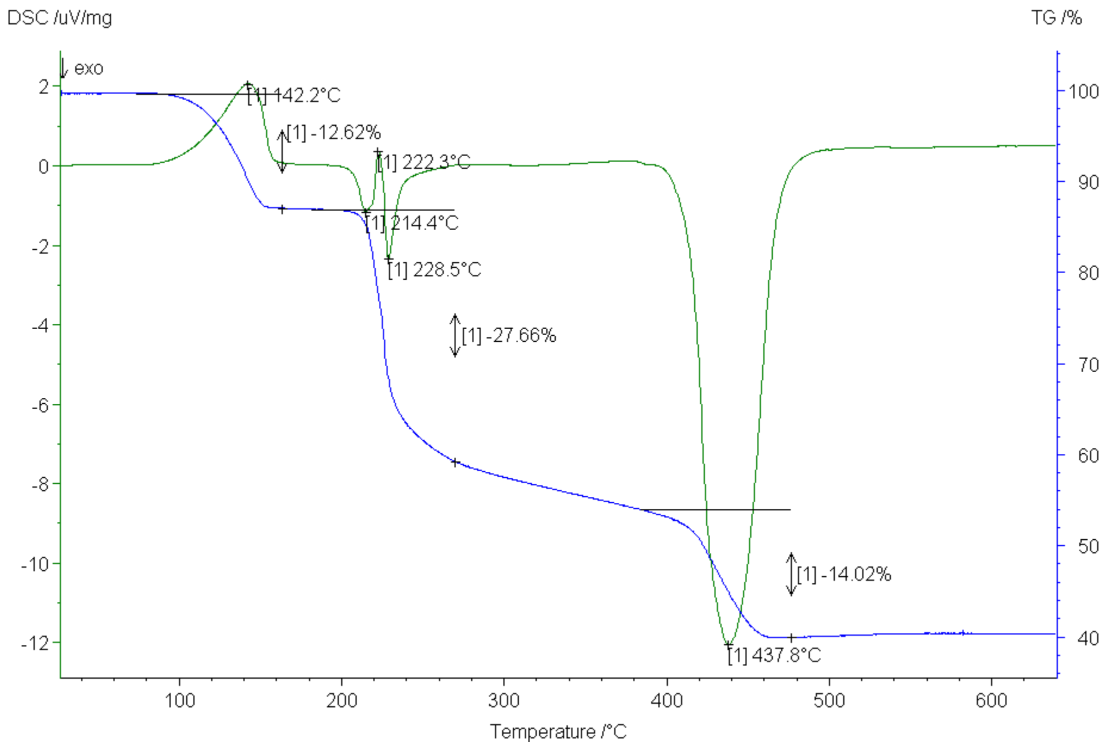

Figure S16. DSC and TGA spectra of CuO NPs treated with Glu.

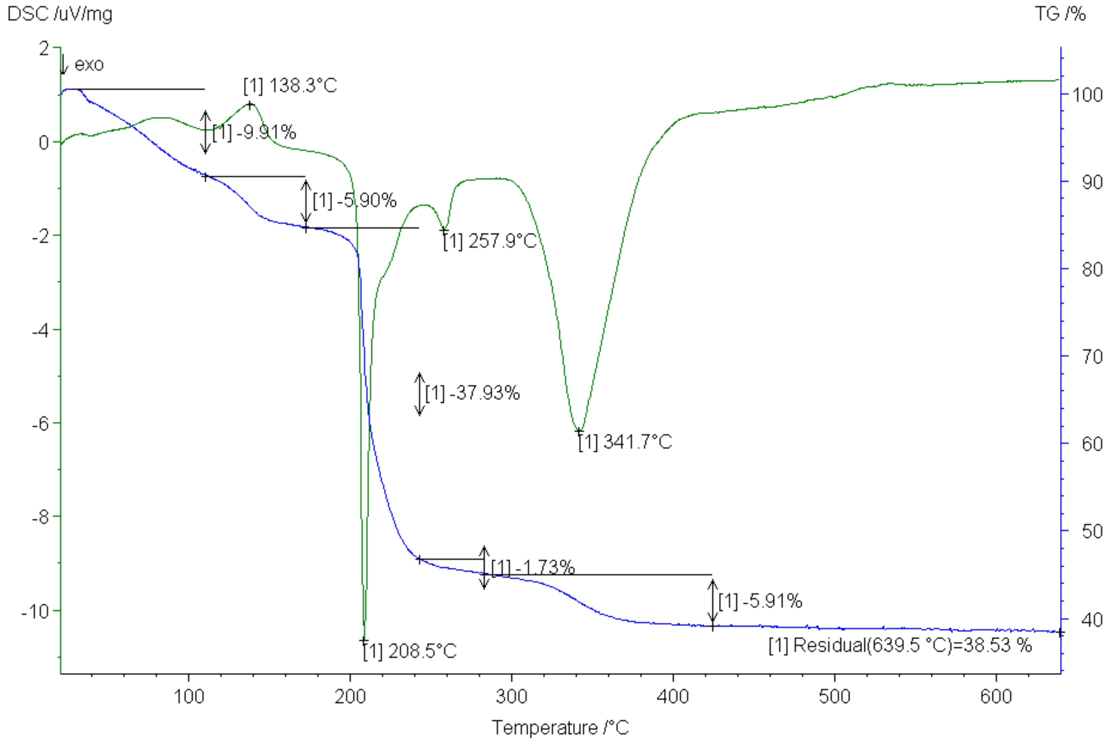

Figure S17. DSC and TGA spectra of CuO NPs treated with Asp.

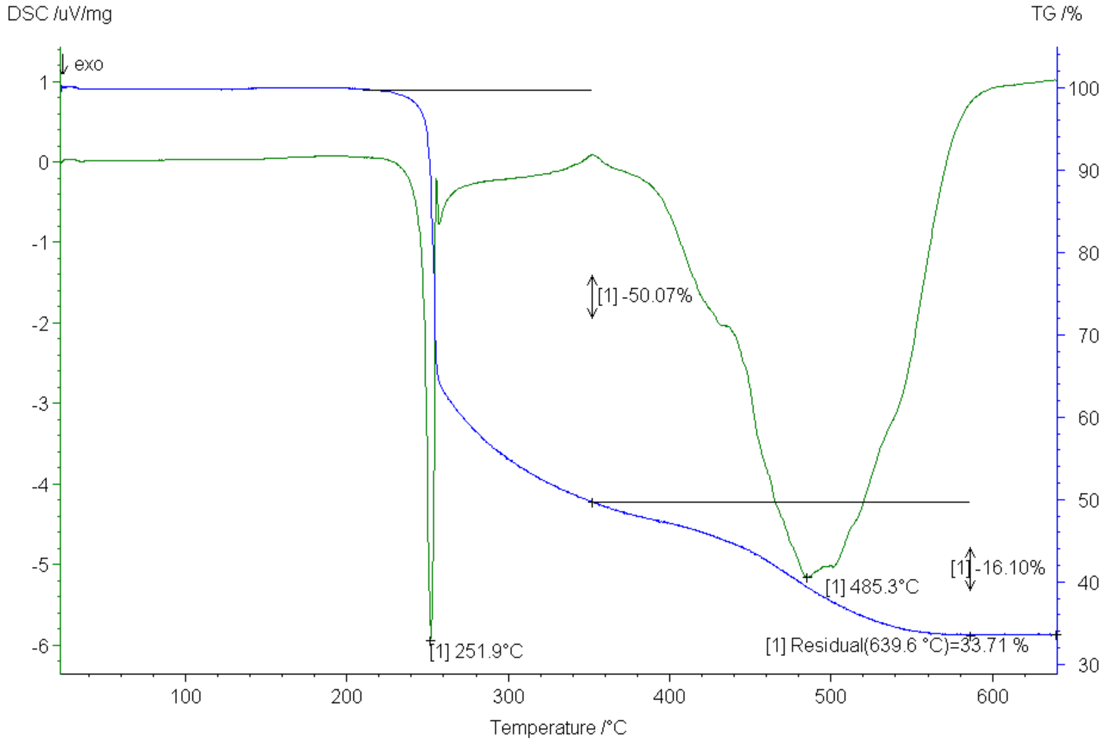

Figure S18. DSC and TGA spectra of CuO NPs treated with Phe.

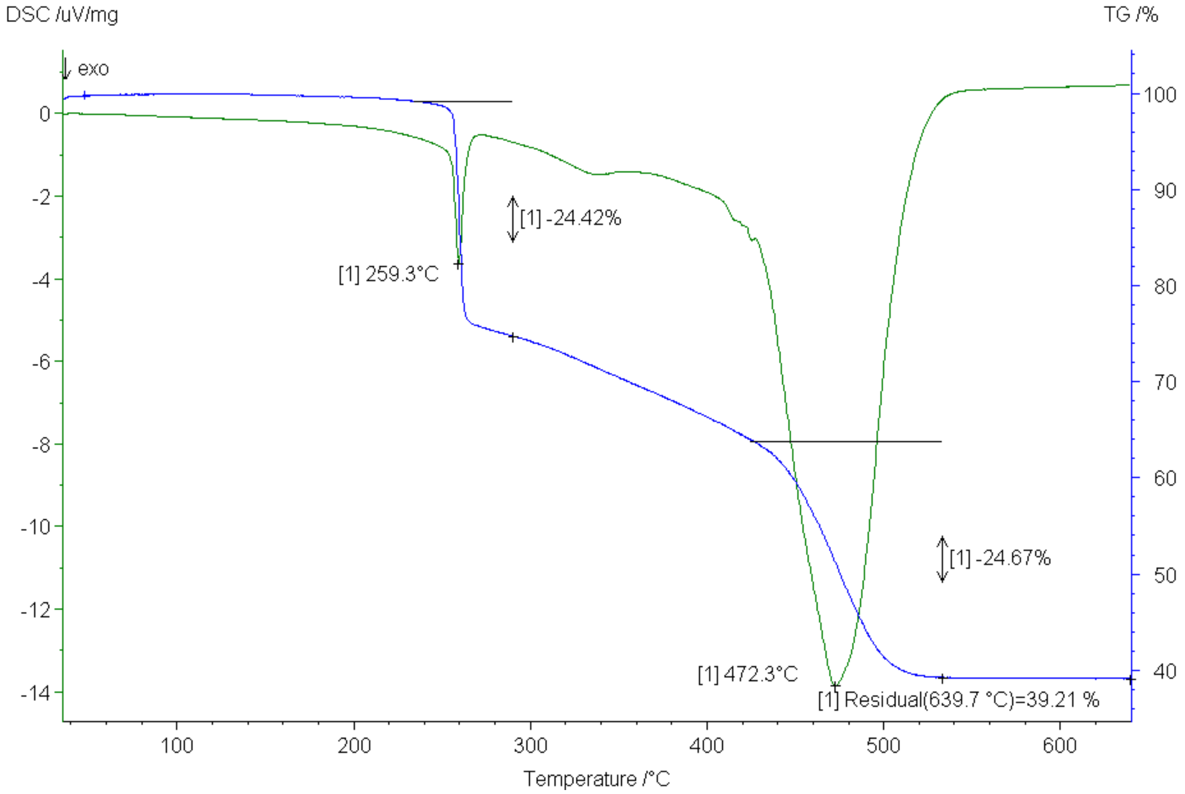

Figure S19. DSC and TGA spectra of CuO NPs treated with Tyr.

Table S2. TG and DSC analysis results.

Compound	Temperature range (°C)	TG weight loss/gain (%)	DSC peaks (°C)	Assignment	Residual mass at 600°C
CuO NPs	30–600	−2.1	-	No reaction occurs	97,9%
CuO NPs+Arg	100–450	−0.7	212, 366 exo, very weak	Combustion of residues remained after cleaning	99.3%
CuO NPs+Val	30–260	−2.1	200 exo, weak	Double step combustion of residues remained after cleaning	95.9%
	410–560	−0.6	515 exo, weak		
CuO NPs+Cys	30–140	−4.4	80, endo	Broad DSC peak centred at 80 °C due to humidity loss	82.8%
	140–200	−10.7	194 exo	1st step of decomposition	
	200–270	−15.9	245 (shoulder) + 270 exo	2nd step of decomposition	
	270–360	+12.4	310 exo	Oxidation	
CuO NPs+Leu	200–270	−56.9	248+272 exo	Double step of decomposition	39.2%
	270–300	+2.16	310 exo (weak)	Oxidation	
CuO NPs+Glu	80–165	−12.9	142 endo	Loss of crystal water	40.4%
	200–270	−27.4	214+229 exo	1st decomposition in a double step reaction	
	400–550	−9.8	438 exo	Combustion of the organic residue	
CuO NPs+Asp	30–110	−9.9%	91 endo	Non-bonded water	38.5%
	110–160	−5.9%	138 endo	Loss of crystal water	
	170–270	−39.6	209+225 exo	Double step decomposition	
			258 exo	Phase transition	
	270–400	−5.9	342	Decomposition	
CuO NPs+Phe	200–270	−40.3	250 exo	Decomposition	33.7%
	400–550	−12.6	425+490 exo	Double step decomposition	
CuO NPs+Tyr	200–270	−24.2	259 exo	Decomposition	39.2%
	270–400	−10.3	334 exo	Decomposition	
	400–550	−27.3	470 exo	Decomposition	

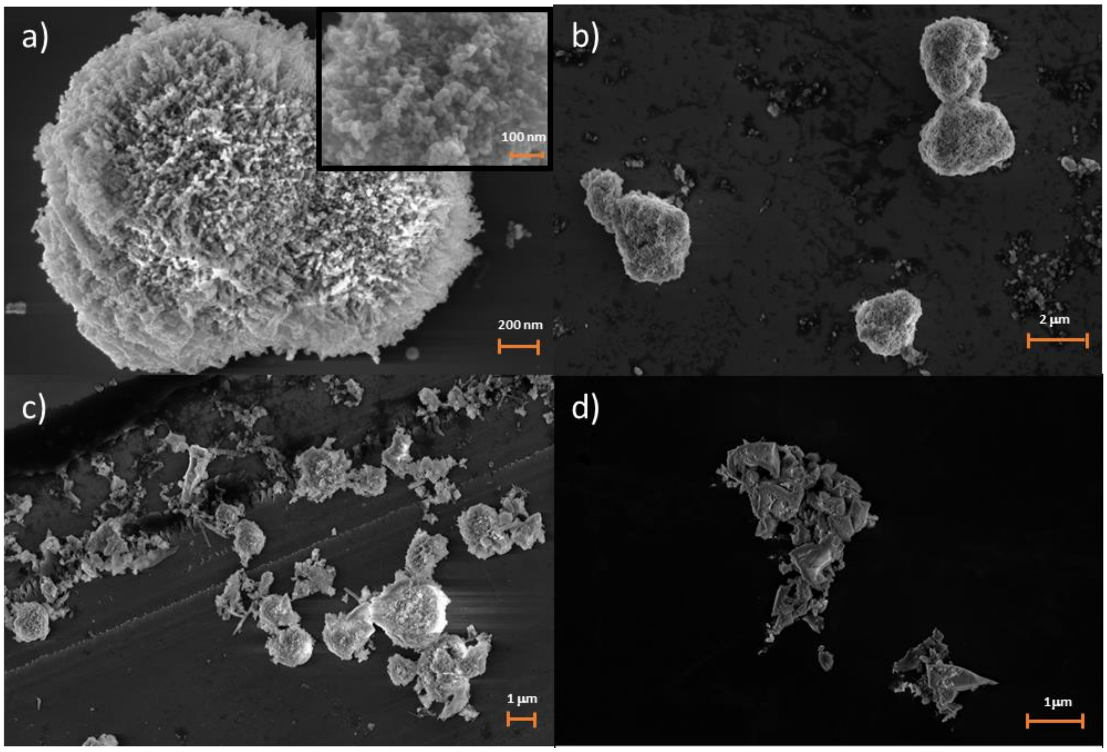

Figure S20. SEM images of (a) pristine CuO NPs; (b) CuO NPs treated with Val; (c) CuO NPs treated with Cys; (d) CuO NPs treated with Tyr.

References:

1. Barth, A. The infrared absorption of amino acid side chains. *Prog. Biophys. Mol. Biol.* 2000, *74*, 141–173.

2. Dokken, K.M.; Parsons, J.G.; McClure, J.; Gardea-Torresdey, J.L. Synthesis and structural analysis of copper(II) cysteine complexes. *Inorganica Chim. Acta* **2009**, *362*, 395–401.

3. Fitts, J.P.; Persson, P.; Brown, G.E.; Parks, G.A. Structure and Bonding of Cu(II)–Glutamate Complexes at the γ-Al2O3–Water Interface. *J. Colloid Interface Sci.* **1999**, *220*, 133–147.

4. Marcu, A.; Stanila, A.; Rusu, D.; Rusu, M.; Cozar, O.; David, L. Spectroscopic studies of copper (II) complexes with some amino acids. *J. Optoelectron. Adv. Mater.* **2007**, *9*, 741–746.

5. Stanila, A.; Marcu, A.; Rusu, D.; Rusu, M.; David, L. Spectroscopic studies of some copper(II) complexes with amino acids. *J. Mol. Struct.* **2007**, *834–836*, 364–368.

6. Silverstein, R.M.; Bassler, C.G.; Morrill, T.C. Spectrometric identification of organic compounds. **1981**, 128–129.
